# A 30-Year Review on Nanocomposites: Comprehensive Bibliometric Insights into Microstructural, Electrical, and Mechanical Properties Assisted by Artificial Intelligence

**DOI:** 10.3390/ma17051088

**Published:** 2024-02-27

**Authors:** Fernando Gomes Souza, Shekhar Bhansali, Kaushik Pal, Fabíola da Silveira Maranhão, Marcella Santos Oliveira, Viviane Silva Valladão, Daniele Silvéria Brandão e Silva, Gabriel Bezerra Silva

**Affiliations:** 1 Biopolymers & Sensors Lab., Instituto de Macromoléculas Professora Eloisa Mano, Universidade Federal do Rio de Janeiro, Centro de Tecnologia-Cidade Universitária, Rio de Janeiro 21941-853, Brazil; drfabiola.sma@gmail.com (F.d.S.M.); marcellasanttos@ima.ufrj.br (M.S.O.); viviane.valladao@ima.ufrj.br (V.S.V.); gabrielbsilva@ima.ufrj.br (G.B.S.); 2 Programa de Engenharia da Nanotecnologia, Instituto Alberto Luiz Coimbra de Pós-Graduação e Pesquisa de Engenharia (COPPE), Universidade Federal do Rio de Janeiro, Centro de Tecnologia-Cidade Universitária, Rio de Janeiro 21941-914, Brazil; danielesb29@gmail.com; 3 Biomolecular Sciences Institute, College of Engineering & Computing, Center for Aquatic Chemistry and Environment, Florida International University, 10555 West Flagler St EC3900, Miami, FL 33174, USA; 4 Department of Physics, University Center for Research and Development (UCRD), Chandigarh University, Mohali 140413, Punjab, India; kaushikphysics@gmail.com

**Keywords:** nanocomposites, bibliometric analysis, sentiment analysis, microstructural characterization, computational methodologies, Scopus database, Boolean search, crosslinking time

## Abstract

From 1990 to 2024, this study presents a groundbreaking bibliometric and sentiment analysis of nanocomposite literature, distinguishing itself from existing reviews through its unique computational methodology. Developed by our research group, this novel approach systematically investigates the evolution of nanocomposites, focusing on microstructural characterization, electrical properties, and mechanical behaviors. By deploying advanced Boolean search strategies within the Scopus database, we achieve a meticulous extraction and in-depth exploration of thematic content, a methodological advancement in the field. Our analysis uniquely identifies critical trends and insights concerning nanocomposite microstructure, electrical attributes, and mechanical performance. The paper goes beyond traditional textual analytics and bibliometric evaluation, offering new interpretations of data and highlighting significant collaborative efforts and influential studies within the nanocomposite domain. Our findings uncover the evolution of research language, thematic shifts, and global contributions, providing a distinct and comprehensive view of the dynamic evolution of nanocomposite research. A critical component of this study is the “State-of-the-Art and Gaps Extracted from Results and Discussions” section, which delves into the latest advancements in nanocomposite research. This section details various nanocomposite types and their properties and introduces novel interpretations of their applications, especially in nanocomposite films. By tracing historical progress and identifying emerging trends, this analysis emphasizes the significance of collaboration and influential studies in molding the field. Moreover, the “Literature Review Guided by Artificial Intelligence” section showcases an innovative AI-guided approach to nanocomposite research, a first in this domain. Focusing on articles from 2023, selected based on citation frequency, this method offers a new perspective on the interplay between nanocomposites and their electrical properties. It highlights the composition, structure, and functionality of various systems, integrating recent findings for a comprehensive overview of current knowledge. The sentiment analysis, with an average score of 0.638771, reflects a positive trend in academic discourse and an increasing recognition of the potential of nanocomposites. Our bibliometric analysis, another methodological novelty, maps the intellectual domain, emphasizing pivotal research themes and the influence of crosslinking time on nanocomposite attributes. While acknowledging its limitations, this study exemplifies the indispensable role of our innovative computational tools in synthesizing and understanding the extensive body of nanocomposite literature. This work not only elucidates prevailing trends but also contributes a unique perspective and novel insights, enhancing our understanding of the nanocomposite research field.

## 1. Introduction

Nanocomposites have become a central focus of scientific inquiry, extending their influence across various sectors, evidenced by the significant research contributions of several authors [1,2,3,4,5] and others, highlighting the transformative potential of these materials. The works of Andritsch [6] and Faulkner [7], among others, have been instrumental in showcasing how advanced manufacturing techniques are crucial for optimizing the performance and functionality of nanocomposites, emphasizing the need to improve processing methods for better structural integrity and interfacial adhesion [6,8,9,10].

A critical area of this research involves the integration of diverse nanofillers, such as carbon nanotubes (CNTs) [11], graphene oxide (GO) [12,13], silica nanoparticles [14,15], and various metal oxides [16,17], into polymer matrices [18,19,20,21,22,23,24,25,26,27,28,29,30,31,32,33,34,35,36,37,38,39,40,41,42,43]. This integration is vital in enhancing the performance of composite materials. The study of nanocellulose composites, in particular, has unveiled their exceptional properties, including strength, renewability, and lightness [44,45,46]. The exploration of new nanofillers and their incorporation into polymers, demonstrated by researchers like Fu et al. [4] and Tian et al. [47], has pushed the boundaries of nanocomposite technology. Moreover, understanding the dispersion of nanocomposites and its effect on material properties offers deep insights, facilitating the development of predictive tools and optimization strategies [48,49,50,51].

The exploration of the nanoscopic world has brought nanocomposites to the forefront of research, signifying a breakthrough in multiple domains, including environmental recovery [23,34,52,53,54,55,56,57,58,59,60,61,62,63,64,65,66,67,68,69,70], electronics [71,72,73,74,75,76,77,78,79,80,81], energy storage [82,83,84,85,86,87,88,89,90,91,92,93,94,95,96,97,98,99,100,101], and healthcare [102,103,104,105,106,107,108,109,110,111,112,113,114,115,116,117,118,119,120]. These nanocomposites, which are composed of diverse materials such as metals, ceramics, and polymers, exemplify the capability to tailor-make materials with specific attributes for a broad range of uses [121,122,123,124].

However, the development of nanomaterials faces considerable challenges. Enhancing mechanical [22,36,40,41,60,67,70,125,126,127,128,129,130,131,132,133,134,135,136,137,138,139,140,141,142,143,144,145,146,147,148,149,150,151,152,153,154,155,156,157,158,159,160,161], electrical [21,27,30,31,32,35,42,55,59,120,160,161,162,163,164,165,166,167,168,169,170,171,172,173,174,175,176,177,178,179,180,181,182,183,184,185,186,187,188,189,190,191,192,193,194,195,196,197,198,199,200,201,202,203], and microstructural [19,53,63,68,118,204,205,206,207,208,209,210,211,212,213,214,215,216,217,218,219,220,221,222,223,224,225,226,227,228,229,230] attributes in nanocomposites requires precise nanoscale manipulation. This is coupled with issues like reproducibility, cost, and complexity in preparation, adding to the intricacies of nanomaterial research [231,232,233,234,235,236,237,238,239,240,241,242,243,244,245,246,247,248,249,250]. As nanotechnology evolves, addressing these technical and ethical issues [251,252,253,254,255,256,257,258,259,260,261,262,263,264,265,266,267,268,269,270,271,272,273,274,275,276] is crucial for maximizing the potential of nanomaterials and ensuring their responsible application in various fields [277,278,279,280].

The advancement of nanomaterials can be expedited by leveraging data mining, an essential tool in scientific research, given the vast and complex data generated. This process is crucial for the progression of nanomaterials [281,282,283,284,285,286].

Incorporating data mining into nanomaterials research aids in identifying new patterns, trends, and relationships in extensive datasets [287,288,289,290,291,292,293,294,295,296,297,298,299]. This is key to discovering novel properties of nanomaterials, predicting material behaviors, and guiding the creation of new nanocomposites with desired characteristics. Data mining also helps map the research landscape in nanomaterials, pinpointing knowledge gaps and suggesting potential areas for further research [216,217,300,301,302].

Nevertheless, data mining brings forth significant concerns, including privacy and security. In this significant data era, maintaining confidentiality and data integrity is vital for the credibility of scientific research. Ethical issues and potential biases in data mining processes also need attention, as biases in data handling can lead to skewed and unjust outcomes [303,304,305,306,307,308,309].

Furthermore, the interpretability and transparency of data mining results are crucial for building trust among stakeholders and understanding complex data analyses. The challenges of data quality and preprocessing are also significant, as inadequate data handling can significantly impact data mining outcomes [310,311,312,313,314,315,316].

Machine learning (ML) is becoming a critical tool in various fields of nanomaterials research, such as nanoindentation [317,318,319,320,321,322,323,324,325,326,327,328], nanorobotics [329,330,331,332,333,334], and nanosensor [335,336,337,338,339,340] development. Its ability to analyze and interpret complex patterns from large datasets is particularly beneficial in advancing areas like nanostructured materials analysis, nanoscale manufacturing processes, and the development of nanotechnology applications in medicine and environmental monitoring [327,341,342,343,344,345,346,347,348,349,350,351,352,353,354,355,356,357,358,359,360,361,362,363,364,365,366,367,368,369,370,371,372,373,374,375,376,377,378,379,380,381,382,383,384,385,386,387,388,389,390,391,392,393,394,395,396,397,398,399,400,401,402,403,404]. Employing unsupervised ML techniques on databases like Scopus, Web of Science, Scielo, and Google Scholar can substantially contribute to nanomaterials research [354,405,406,407,408,409,410,411,412,413,414,415,416,417,418,419,420,421,422,423,424,425,426,427,428,429,430,431,432,433,434,435,436]. These techniques autonomously analyze large volumes of unstructured data, identifying hidden patterns and correlations essential for uncovering new material properties, understanding nanoscale interactions, and predicting material behaviors [140]. This facilitates the synthesis of novel nanocomposites and the optimization of their properties for diverse applications [425,437,438,439].

This paper conducts a systematic and comprehensive review of the nanocomposite research landscape, focusing on microstructure, electrical properties, and mechanical behavior. It utilizes insights from the Scopus database, combining textual and bibliometric analysis to provide a detailed overview of the research field.

Given the immense complexity and volume of data from sources like Scopus, traditional analytical methods often fall short. Thus, this study employs computational tools, including data mining and ML techniques like Boolean searches and Latent Dirichlet Allocation (LDA) [419,440], to navigate and interpret the vast data effectively, revealing key themes in nanocomposite research.

The research landscape is refined using network graph methodologies to identify key research nodes and their interconnections [31,120,140,215,217,218,301,302,441,442,443,444,445,446,447,448,449,450,451], spotlighting pivotal works and active research clusters, especially in emerging research hubs like the United States, China, and India.

Advanced textual analysis, akin to platforms like Voyant Tools but customized for this study, uses natural language processing (NLP) to dissect academic content meticulously [452,453,454,455,456,457,458,459,460,461,462,463,464]. This aligns with the core themes of microstructural characterization, electrical dynamics, and mechanical complexities in nanocomposites.

This study incorporates a detailed bibliometric analysis, mapping the pivotal contributions and key thought leaders in nanocomposites. This comprehensive approach provides an in-depth understanding of the foundational aspects of the field. It anticipates emerging trends, such as incorporating machine learning (ML) and Artificial Intelligence (AI) in designing and optimizing nanocomposites.

The research highlights critical areas that require further investigation, including aspects related to nanocomposites’ durability, stability, and potential toxicological impacts. Despite promising results in laboratory settings, scalability and commercial viability challenges persist. The study also notes a marked increase in research focused on energy storage solutions, such as high-performance batteries and supercapacitors, which utilize nanocomposite technologies, underscoring the dynamic progression of this field.

A combined bibliometric and sentiment analysis of nanocomposite literature is also undertaken, revealing a gradual shift toward more positive sentiment in academic abstracts. This shift suggests improvements in how researchers communicate their findings or reflects an increasing enthusiasm for breakthroughs in nanocomposite research.

Our study expands upon traditional reviews by integrating a novel computational approach to organize research within the nanomaterials field. We respect the profound insights of conventional reviews, which draw from the rich knowledge of experienced authors. However, our methodology is designed to complement, rather than replace, such expertise. It serves as a resource for researchers, especially those newer to the field, to swiftly identify key and emerging topics within nanocomposite research, offering a tool for rapidly pinpointing crucial and novel areas within nanocomposite studies. The computational tools we have developed facilitate a more effective and thorough exploration of the scientific landscape, particularly in identifying trends and insights that may not be immediately apparent through conventional methods alone.

Our analysis of “Microstructural, Electrical, and Mechanical Properties” exemplifies this methodology, showcasing how computational advancements can accelerate the understanding and development of nanomaterials. As such, this paper contributes to the existing body of knowledge and presents a pioneering approach to research synthesis in nanomaterials.

## 2. Methodology

The computational analyses in this study were conducted using a high-performance workstation provided by Tyar, located in São Paulo, SP, Brazil. The system features a 13th Gen Intel® Core™ i9-13900KF processor, 62.6 GiB of RAM, and an NVIDIA GeForce RTX3080 graphics card.

### 2.1. Scopus Database Search Strategy

On 23 October 2023, a detailed search of the Scopus database identified key research areas related to nanocomposites, focusing on their microstructural, electrical, and mechanical characteristics. The search parameters encompassed titles, abstracts, and keywords of articles from all publication dates, used Boolean search techniques with three specific queries: “Nanocomposi*” and “Microstructure”, “nanocomposi*” and “Electrical properties”, and “Nanocomposi*” and “Mechanical behavior”. This approach facilitated the identification of a clear view of current research trends in nanocomposites, particularly in fundamental characterization. In the following sections, we analyze these data, highlighting emerging trends and key findings from the research articles.

### 2.2. Textual Analytics Approach

As stated earlier, this study used proprietary software similar to Voyant Tools for advanced text analytics, focusing on microstructural characterization, electrical properties, and mechanical behavior of nanocomposites in the academic literature. Employing natural language processing (NLP), the software refined data, filtered irrelevant terms, and visually presented results. It integrated data into pandas DataFrames and used NLTK modules for text processing. It engaged key NLTK modules, such as PorterStemmer and word tokenizer, for text processing.

The software identified and quantified frequent terms, enabling us to specify the number of terms for analysis or defaulting to the top 10. It also performed temporal data analysis, generating year-specific CSV files and visualizing annual term significance trends. This tool provided insights into the nanocomposite research landscape, emphasizing thematic perspectives.

### 2.3. Scholarly Literature Analysis on Nanocomposite Themes

We developed a custom-developed software rooted in Python 3.8.10 and leveraged libraries such as pandas, NLTK, Gensim, and Matplotlib. The resulting software excelled in tracking the evolution of scholarly literature, conducting sentiment analysis on titles and abstracts, precise topic modeling, and intricate visual representation.

The software starts with data import and refinement and then seamlessly integrates metadata from relevant scholarly articles in CSV format, including parameters like title, abstract, publication year, authors, and publishers. It filters out records with incomplete titles or abstracts, ensuring a high-quality dataset for a thorough review.

Next, the software employs sci-kit-learn methodologies to analyze and visually represent data points such as annual publication counts, author contributions, and the spectrum of publishing entities, enabling us to visualize the historical development of niche domains within nanocomposite themes.

The platform transitioned to thematic modeling using the Latent Dirichlet Allocation (LDA) algorithm. It analyzed titles and abstracts to uncover prominent themes and keywords in nanocomposite literature, highlighting key discourses in this extensive field.

Visualization played a crucial role in the software’s capabilities, creating word clouds that reflected identified themes from LDA analysis. These word clouds, along with structured graphical representations, provided layered insights into published works.

Additionally, the software facilitated the generation of comprehensive reports that included textual explanations, tabulated insights, and graphical illustrations, offering readers a comprehensive view of the academic landscape specific to nanocomposite domains.

### 2.4. Analysis of MAP and NET Files

The analysis began using VOSviewer version 1.6.19, focusing on content extracted from titles and abstracts. Three RIS files served as the foundational datasets. Following the application of a minimum occurrence threshold equal to 3, a total of 29,843 terms were selected and evaluated in this analysis, encompassing all available terms, were selected. Additional details on this analytical tool can be found in a previous study [217]. 

Next, the authors deployed their custom-designed software, precisely engineered to process and visually represent data extracted from VOSViewer MAP and NET files. For a comprehensive exploration of the RIS files, a repository has been provided and made available on GitHub (https://github.com/ftir-mc/Nano-MEM, accessed on 25 October 2023).

The software follows a structured workflow, beginning with setup and dependency verification. In cases of missing libraries, it autonomously sources and integrates them for operational efficiency. The user-friendly interface, developed using the Tkinter library, offers an intuitive environment for selecting MAP and NET files for analysis, with transparent directory displays.

Data processing follows data import, with essential libraries in action. The software efficiently imports data from the MAP and NET files into data frames, refining MAP data for clarity. Column designations are optimized for enhanced understanding, and data are organized by parameters such as cluster and Total Link Strength (TLS), with relationships visualized and archived. Additional columns, such as TLS_per_Occ, are introduced based on computational determinants.

In the data organization phase, new columns facilitate in-depth analysis. The primary data frame undergoes segmentation based on variables like cluster and publication year. Salient nodes representing each cluster are systematically extracted and preserved for future analysis.

Visualization is crucial, with the software creating treemaps illustrating node distribution across clusters and bar diagrams highlighting predominant nodes. Enhanced treemaps provide insights into network datasets’ intricacies.

The software also conducts extended analysis, including calculating Euclidean distances between nodes, organizing NET data to establish meaningful associations, and applying Tukey’s test to identify statistical variances in annual averages among clusters.

All analytical insights, from detailed cluster breakdowns to empirical statistical interpretations, are consolidated into an extensive textual report, serving as a valuable academic reference.

The software includes supplementary features, such as visual displays of random data matrices using Matplotlib, customization of Excel outputs with conditional formatting, and generating a DOCX document consolidating key metrics to enhance users’ understanding of data significance.

In summary, this methodology showcases the capabilities of the authors’ custom software. Equipped with an intuitive interface and a range of data processing, visualization, and analytical tools, this software is a valuable asset for researchers aiming to extract profound insights from VOSViewer MAP and NET files, particularly in materials science and data mining domains.

### 2.5. Bibliometric Data Analysis and Visualization Report

This section outlines the methodology used for bibliometric data analysis and visualization, achieved through a custom software tool created by the authors. The software effectively integrates functionalities from prominent Python libraries to reveal relationships among research keywords.

Starting with data collection and preprocessing, the software operates on a dedicated CSV file containing the required bibliometric data. It selectively targets keywords: “.nanocomp.” and “.lectrical propert.” Additionally, two further combinations were employed: “.nanocomp.” and “.echanical behav.” and “.nanocomp.” and “microstruct”. Using the pandas library, it imports and refines the dataset to include only records associated with these keywords.

Data segmentation follows, with the curated dataset categorized into four distinct groups based on criteria such as Link Strength Between Items or Terms (LSBI), average publication years for the source and target terms (“year_i” and “year_f”), and Euclidean distance (“DE”) between the source and target terms. Segmentation criteria are determined by the top or bottom ten entries within these columns.

Hierarchical clustering plays a central role in the analysis, employing methodologies from the scikit-learn library to group similar data points. The optimal number of clusters is determined using the Elbow Method, enabling the labeling of clusters within the dataset.

The analysis then proceeds to generate network graphs. Each segmented dataset is represented through network graphs created using the NetworkX 3.2.1 library. Keywords are depicted as nodes, and their interconnections as edges. Node size corresponds to the frequency of the associated keyword, providing insights into keyword significance.

To enhance clarity, graph visualization incorporates elements such as color differentiation based on cluster labels and edges illustrating Link Strength Between Items (LSBI) and Euclidean distance (ED) between terms. The Matplotlib library ensures vivid and comprehensible graph displays, with options to save visual representations in PNG format.

Data tabulation is integral, with network graphs translated into DataFrame structures and stored in CSV format for further analysis.

The synthesis of findings culminates in a comprehensive report generated with the Python-docx library. This report includes an overview of processes, detailed breakdowns of each graph, and an extensive data exploration, focusing on selected keywords. Key findings, including those from the Elbow Method and cluster sizes related to keyword frequencies, are highlighted.

The software significantly enhances analysis by applying hierarchical clustering techniques to bibliometric data, processing both MAP and NET file formats, and consolidating data within a single spreadsheet. User-defined keyword searches enable researchers to explore intricate relationships between keywords and other essential terms.

The software concludes by presenting enhanced visualization and analysis, offering a sequence of network graphs visually depicting keyword interconnections. These graphs include essential data metrics, such as LSBI values, cluster identifiers, average publication years, and term distances, with customization options based on keyword priorities.

In summary, the custom software developed by the authors serves as a powerful tool for exploring and visualizing bibliometric data. Leveraging clustering and network analysis empowers researchers to extract valuable insights, enhancing their understanding of the research domain. This software was employed for all three combinations of nanocomposites with microstructure, mechanical behavior, or electrical properties, forming the basis of the presented review paper.

Supplementary Figure S1 serves as a visual guide to enhance comprehension of the methodological sequence employed in our study. This diagram meticulously outlines each methodology step, from the initial Scopus Database Search Strategy to the final stages of bibliometric data analysis and visualization. Additionally, the figure seamlessly integrates the sequence of results derived from these methods. By illustrating the connections between various methodological steps and their corresponding outcomes, the diagram aids in understanding how each phase of the research contributes to the overall findings and conclusions of the study. This visual representation simplifies the complex processes involved and highlights the logical flow and interrelation of the methods and results in our comprehensive nanocomposite research.

## 3. Results and Discussions

The Scopus database, a prominent repository of scholarly articles, offers valuable insights into research trends. As of 23 October 2023, a comprehensive review of nanocomposites reveals distinct thematic distributions. Although a general nanocomposites search provides broad insights, additional focus on the microstructural, electrical, and mechanical characterization of nanocomposites uniquely showcases our software ability when investigating with a narrow focus.

Upon analysis, the theme centered on “Nanocomposi*” AND “Microstructure” yielded 13,317 documents, representing approximately 6.36% of all nanocomposites research. Exploring electrical properties through “Nanocomposi*” AND “Electrical properties” produced 5370 documents, accounting for about 2.57% of the overall nanocomposites documentation. Additionally, the theme of mechanical behavior, defined by “Nanocomposi*” AND “Mechanical behavior”, revealed 2011 documents, constituting roughly 0.96% of the total nanocomposites research corpus.

When comparing these results to the extensive pool of nanocomposite articles totaling 209,012 documents, several insights emerge. Microstructural studies in nanocomposites form a substantial portion, emphasizing the significance of understanding microscopic organization and its correlation with material properties. Electrical properties, although slightly fewer in volume than microstructural studies, remain significant, shedding light on the critical electrical attributes of these composite materials. Lastly, mechanical behavior, occupying a smaller segment among the three thematic areas, remains pivotal, especially when evaluating how nanocomposites respond to various stress conditions.

In conclusion, this analysis highlights that while the field of nanocomposite research is vast and multifaceted, thematic dissection provides a clearer understanding of specific areas of emphasis. The data obtained through this effort underscore the dynamic trajectory of nanocomposite research, emphasizing the importance of microstructure, electrical properties, and mechanical behavior in the ongoing academic discourse.

### 3.1. Scopus Database Search Strategy

**Figure 1:**
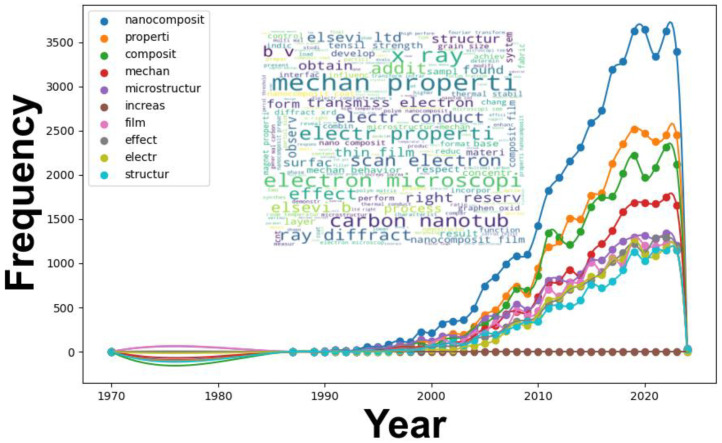
Scatter plot of word frequencies and word cloud of most common words as figures’ inset.

Analyzing the Scopus database reveals central themes in nanocomposite research, as illustrated in Figure 1.

From the late 1970s to the 1990s, the term “nanocomposite” was relatively infrequent, indicating the early stages of this field. However, a notable increase in usage emerged in the mid-1990s, aligning with the growing importance of nanotechnology. In the 21st century, we have witnessed a significant surge in this term, reaching its peak between 2018 and 2022, followed by a slight decline attributed to the incompleteness of the publication data for 2023, as our analysis concluded in October 2023. We anticipate a more comprehensive picture once all publications from 2023 are fully accounted for.

The term “properties” mirrored this trend, reflecting a heightened focus on understanding nanocomposites’ inherent attributes. In contrast, “composite” showed a more gradual rise, indicative of general discussions on composites before the dominance of nanoscale considerations.

Technically, terms such as “mechan”, “microstructure”, and “electr” (likely “electrical”) gained prominence in the 1990s. The emphasis on “microstructure” underscores the importance of understanding the microscale arrangement of nanocomposites. Simultaneously, the prominence of “mechan” and “electr” suggests intensified investigations into the mechanical and electrical properties of nanocomposites.

While “increase” had limited prominence, “film” saw a notable rise from the 1990s, peaking in the 2010s. This likely reflects research on nanocomposite films, which are vital for applications like coatings and barrier materials. The term “effect”, although initially subdued, exhibited consistent growth, indicating interest in understanding the consequences and potential applications of nanocomposites.

The term “structur” (likely “structure”) became more prominent from the late 1980s onward, highlighting the foundational interest in comprehending the structure of nanocomposites and its implications for properties and applications.

The word cloud in Figure 1 visually represents recurring terms in nanocomposite research. For the “Microstructure” theme, the term itself holds prominence, emphasizing its pivotal role. Microstructural characterization aims to provide insights into aspects like arrangement, morphology, and phase distribution. Supplementary terms such as “size”, “layer”, “scan electron”, and “microscopy” underscore the reliance on tools like scanning electron microscopy (SEM) for microstructural elucidation.

In the context of “Electrical properties”, terms like “electron”, “conduct”, and “dielectric” highlight the electrical behavior of nanocomposites. Introducing nanofillers can enhance electrical properties, and the term “dielectric” suggests studies on materials under external electric fields, which are crucial for capacitive and insulative applications.

Concerning “Mechanical behavior”, terms like “mechan”, “strength”, “modulus”, and “resist” are salient. They collectively emphasize efforts to comprehend the mechanical responses of nanocomposites, including their influence on attributes like tensile strength, modulus, and toughness.

In summary, this analysis outlines the multifaceted trajectory of nanocomposites research. The fusion of advanced characterization techniques with data analysis has yielded profound insights into the intricate relationships among microstructure, electrical properties, and mechanical behavior in nanocomposites. The data underscore the dynamic and ever-evolving nature of nanocomposite research.

### 3.2. Textual Analytics Approach

**Figure 2:**
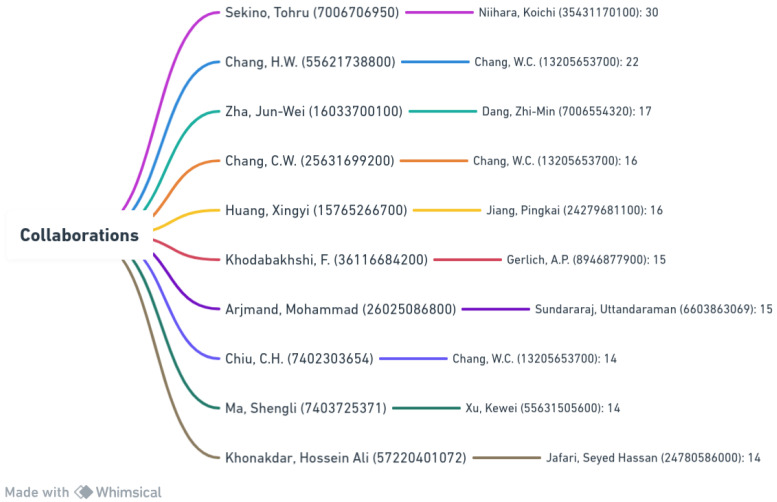
Top ten collaborations between authors. The figure presents the pair of collaborating authors, their Scopus IDs in parentheses, and the number of joined publications.

Figure 2 provides insights into collaborations among scholars in the nanocomposites field. It outlines the top 10 collaborations based on citations, indicating the frequency of joint publications by author pairs. Moreover, the Scopus ID of the author is presented in parentheses. Notably, Sekino, Tohru, and Niihara, Koichi emerged as prominent collaborators, with 30 joint publications, signifying a robust scholarly partnership. Chang, W.C. is also notable for contributing prolifically, highlighting the significance of collaborative research in knowledge dissemination.

**Figure 3:**
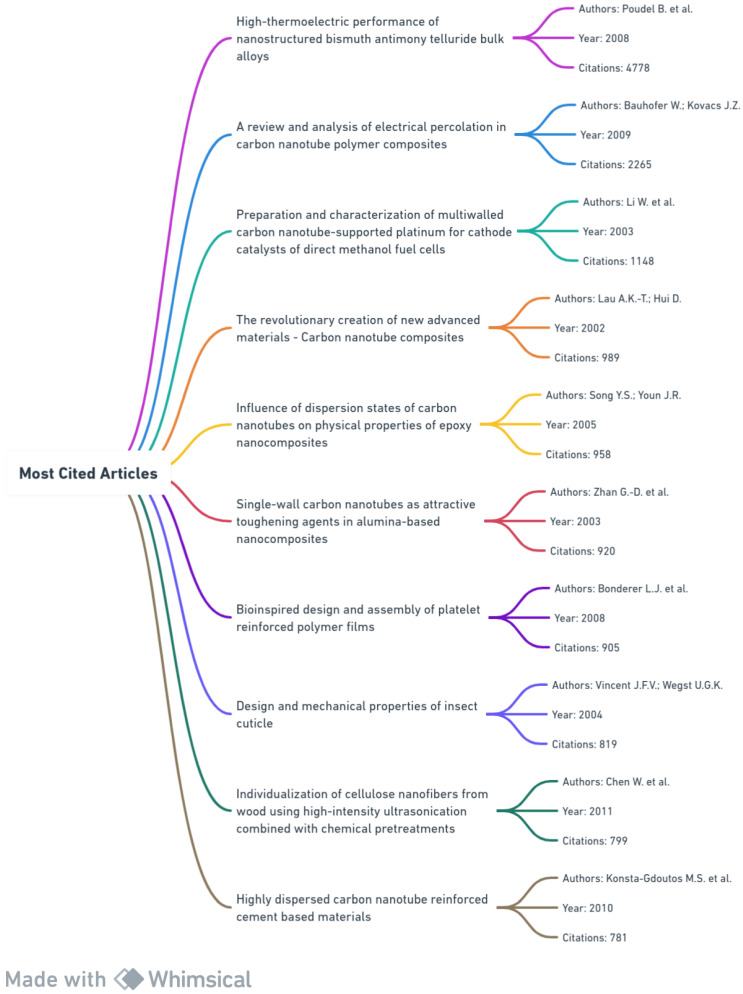
Top ten most cited articles showcasing titles, authors, publication years, and citation counts. which is available at (https://whimsical.com/most-cited-articles-MUUsxarSj8osB2j2C4rK7).

Figure 3 presents the top 10 most cited articles in nanocomposite research, showcasing titles, authors, publication years, and citation counts.

“High-thermoelectric performance of nanostructured bismuth antimony telluride bulk alloys” stands out with 4778 citations, underscoring its seminal impact. The temporal distribution of articles suggests heightened interest in nanocomposites from 2002 to 2011, possibly due to advancements in characterization techniques or recognition of nanocomposites’ potential in various applications. The diverse topics within these articles, from thermoelectric properties to bioinspired design, exemplify the versatility and interdisciplinary nature of nanocomposite research.

**Figure 4:**
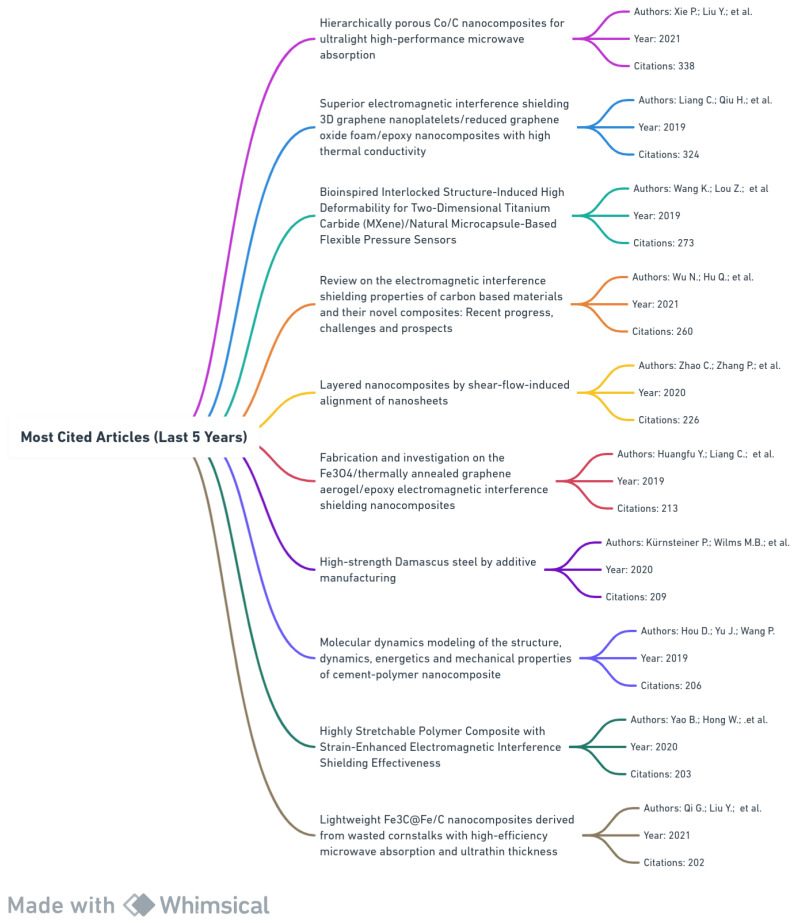
Top 10 most cited articles (last 5 years) showcasing titles, authors, publication years, and citation counts. which is available at (https://whimsical.com/most-cited-articles-last-5-years-4TZqhfjDtnw59ACMVeBZzU).

Figure 4 lists the top 10 most cited articles related to nanocomposites published in the last five years, highlighting critical parameters: title, authors, publication year, and citation count.

Notably, the article by Xie P. et al. 2021 on “Hierarchically porous Co/C nanocomposites for ultralight high-performance microwave absorption” leads with 338 citations, indicative of a resurgence of interest in the nanocomposite field. EMI shielding properties are a recurring theme in the second, fifth, and ninth most cited articles, emphasizing their relevance in modern electronics. The article by Wang K. et al. in 2019 explores bioinspired structures for flexible pressure sensors, reflecting interdisciplinary research trends. Additionally, a 2020 publication by Kürnsteiner P. et al. on “High-strength Damascus steel by additive manufacturing” highlights the fusion of ancient techniques with nanotechnology. Lastly, the work by Qi G. et al. in 2021 emphasizes sustainable research practices using waste materials.

**Figure 5:**
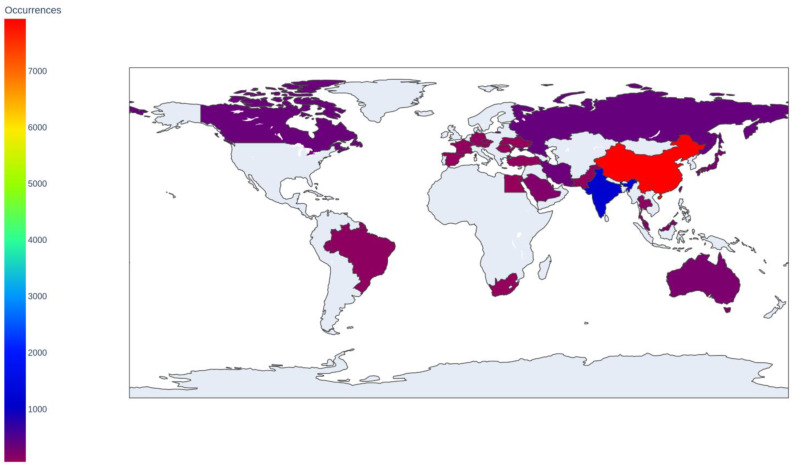
Funding sources by country, indicating the number of occurrences reflecting research investment. The color temperature represents the volume of funding, with warm red signifying the highest investment by China, followed by colder shades for other countries in descending order of their investment occurrences.

Figure 5 reveals distinct patterns in funding sources by country, offering insights into the current state of nanocomposite research globally.

China takes the lead with an impressive 7669 occurrences, showcasing its significant investment in nanocomposite research. This dominance reflects China’s robust commitment to innovation and technological advancement, bolstered by its substantial economy.

India follows as a distant second with 1109 occurrences, signaling its growing presence in the global research community. India’s emphasis on advanced materials aligns with its expanding industrial sector and diverse research interests.

The dataset also highlights contributions from countries across continents. Russia, Canada, and Iran closely follow, with occurrences ranging from 364 to 335, demonstrating a global interest in nanocomposites beyond regional boundaries.

Countries like Taiwan, Japan, Australia, Malaysia, and Saudi Arabia, though with fewer occurrences, make substantial contributions, emphasizing the global recognition of nanocomposites’ significance.

European countries like Austria, Germany, France, Czech Republic, Romania, and Spain also feature, showcasing Europe’s continued commitment to pioneering research.

Notably, countries less commonly associated with global research, such as Qatar, Egypt, and Ukraine, appear, indicating a democratization of research efforts and a more inclusive global research landscape.

**Figure 6:**
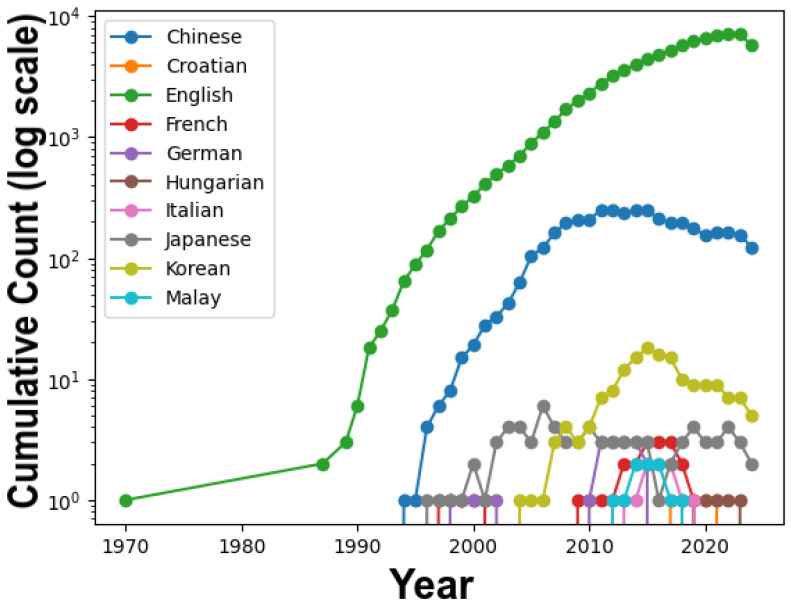
Cumulative language evolution.

Figure 6 illustrates the evolution of academic publications across nine different languages from 1970 to 2020, shedding light on the diverse landscape of research languages.

From 1970 to 1985, English stood as the sole language of academic research, a testament to its historical role as the predominant language in academia and the substantial investments of English-speaking countries in research and development.

In the 1990s, English continued to grow, and Chinese began to make its presence felt in academic research, reflecting globalization trends and China’s increasing economic and technological influence.

The 2000s witnessed a significant surge in English and Chinese publications, driven by globalization, technological advancements, and Asia’s rising prominence in research.

Between 2010 and 2020, the predominance of English in academic publishing subtly diminished, reflecting a broader trend toward linguistic diversity in scholarly communication. Concurrently, the research output in Chinese showed signs of slowing, possibly indicating a shift in research priorities within China. Notably, languages such as Korean, Italian, and Croatian—the latter acknowledged as an independent language since 1991 and included in our analysis due to its distinctive historical and linguistic significance—made their presence known. This diversification emphasizes a gradual move toward inclusivity in the dissemination of academic knowledge, spotlighting the global nature of scientific research and the importance of embracing a variety of linguistic perspectives. This is increasingly enabled by the advent of Large Language Models (LLMs), which facilitate communication across numerous languages, thereby broadening access to knowledge worldwide.

Figure 6 provides a comprehensive view of how academic research languages have evolved over five decades, with implications for broader global socio-economic, technological, and geopolitical transformations.

Additionally, extensive data on countries producing academic articles related to specific chemicals associated with nanocomposites reflect the global landscape of research in this field.

China leads with an astounding 7072 articles where a specific chemical is not declared (notated as “nan”). China’s research is diverse, encompassing chemicals like graphite, water, hydroxyapatite, cellulose, and more, demonstrating a comprehensive approach to nanocomposite research.

India follows with 1035 “nan” articles, focusing on hydroxyapatite, biocompatible materials, graphite, chitosan, and antibacterial agents.

Russia’s research, represented by 349 “nan” articles, emphasizes biocompatible materials, capsules, and magnetite nanoparticles, along with niche chemicals like erythropoietin.

Other countries like Canada, Iran, Taiwan, Japan, Australia, Malaysia, and Saudi Arabia have distinct chemical focuses, reflecting regional interests and industrial applications.

**Figure 7:**
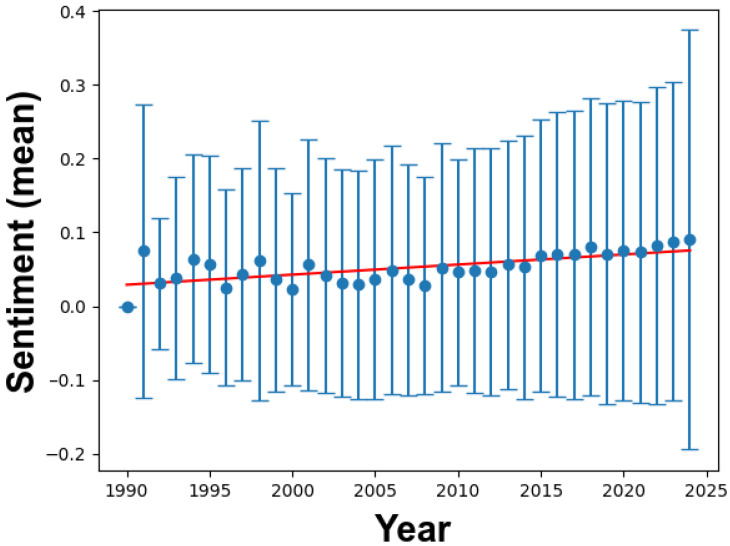
Titles sentiment scatter plot.

Figure 7 portrays the sentiment trends in academic titles from 1990 to 2024, offering insights into evolving academic cultures, research focuses, and potential global influences.

A glance at the scatter plot with error bars reveals a consistent sentiment hovering near neutrality, with slight fluctuations over the years. These error bars, indicating standard deviations, show that while individual titles may vary in sentiment, the overall outlook remains balanced.

The regression line, represented by the equation y = −2.690 + 0.001 x, indicates a subtle positive slope, suggesting a gradual increase in sentiment over time. However, this increase is relatively small, reaffirming the predominantly neutral nature of academic titles.

The R^2^ value of 0.445 indicates that about 44.5% of sentiment variability can be attributed to publication year, emphasizing the influence of other external factors beyond temporal changes.

Further examination in the “Global Statistics for Titles” table reveals that from 1990 to 2024, 19,645 titles were analyzed. The average sentiment across these titles is 0.052343, confirming the prevalence of a neutral tone. The standard deviation of 0.166729 reinforces the consistency in this neutrality.

The general neutrality in academic titles may stem from the objective nature of scientific research, requiring a factual and impartial tone. While major global events or technological advancements may have briefly influenced sentiment, these effects have been balanced by other factors, resulting in a median sentiment trajectory.

**Figure 8:**
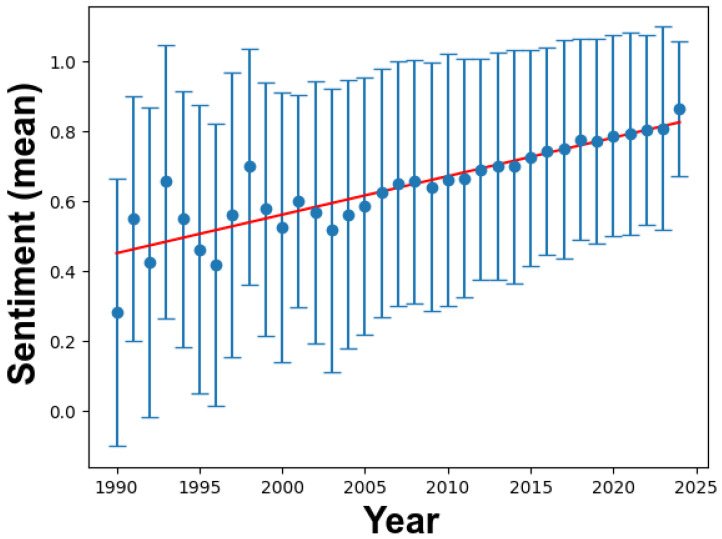
Abstracts sentiment scatter plot.

Figure 8 provides a temporal analysis of sentiment trends in academic abstracts from 1990 to 2024, employing advanced textual analytics to uncover underlying sentiment patterns.

The scatter plot, accompanied by error bars, indicates a consistent sentiment above neutrality throughout the studied period. The error bars represent data variability, highlighting that while abstracts generally maintain a positive sentiment, there are fluctuations from year to year. Some notably long error bars indicate years with more significant sentiment divergence in abstracts.

The regression line, described by y = −21.484 + 0.011 x and boasting an impressive R^2^ value of 0.777, depicts a gentle, positive slope. This suggests a gradual shift toward more positive sentiment over time, indicating that researchers are becoming better at expressing the significance of their work or their enthusiasm for their findings.

Global statistics reveal that among 19,352 abstracts from 1990 to 2024, the mean sentiment is 0.638771, with a standard deviation of 0.343021. The relatively high mean sentiment confirms a preference for positive expression in academic abstracts within this domain. The standard deviation underscores the range of sentiment expressions present in the corpus despite the prevalent positivity.

Comparing titles and abstracts, abstracts exhibit a more pronounced positive sentiment. This difference in sentiment dynamics can be attributed to the nature of these textual elements. While titles are concise summaries, abstracts offer a deeper exploration of methodologies, findings, and implications, allowing authors to convey their results and breakthroughs with more depth and emotion.

In conclusion, sentiment trends in abstracts reflect an environment of progressive optimism and notable breakthroughs in nanocomposite research. This meticulous text analytics approach provides valuable insights into the evolving mindset of the academic community. The increasing positive sentiment trajectory signifies not only advancements in research but also growing confidence in communicating these achievements.

### 3.3. Scholarly Literature Analysis on Nanocomposite Themes

**Figure 9:**
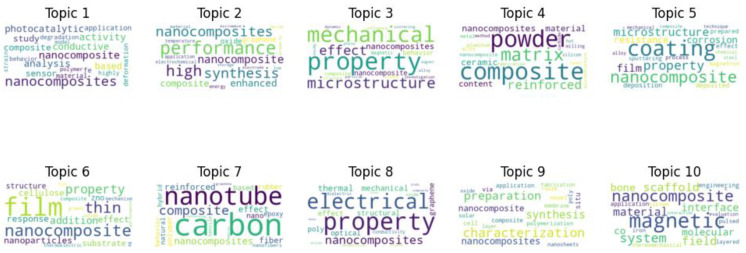
LDA-generated word clouds from titles.

In Figure 9, the data generated through Latent Dirichlet Allocation (LDA) reveal the multifaceted nature of nanocomposite research topics within scholarly literature titles. This algorithmic analysis uncovers the primary thematic directions shaping contemporary discourse in the field.

Title topic 1 centers on the analytical and functional aspects of nanocomposites. Terms like “activity”, “analysis”, and “sensor” highlight a focus on practical applications, particularly in sensing and photocatalysis. The term “conductive” underscores interest in the electronic properties of nanocomposites, suggesting potential in advanced electronics and optoelectronics. Title topic 2 emphasizes synthesis, performance enhancement, and materials like graphene. Research in this area aims to improve nanocomposite performance, often by leveraging exceptional materials like graphene. Title topic 3 delves into the microstructural and mechanical attributes of nanocomposites. Words like “property”, “mechanical”, “microstructure”, and “behavior” signify a keen interest in understanding and manipulating microscale physical characteristics and their influence on overall mechanical behavior. Subsequent topics reveal diverse research directions. Topics 4 and 5 focus on “composite”, “matrix”, and “coating”, indicating research into integrating nanoscale components into matrices or surfaces for improved structural or protective properties. Topic 6 highlights the importance of “film” and “thin” nanocomposite films, applicable in various fields from electronics to coatings. Topic 7 explores carbon nanotubes’ role in composites, emphasizing their reinforcing capabilities. Topic 8 showcases nanocomposites’ multifunctional attributes, including electrical, thermal, and optical properties, reflecting a multidisciplinary approach. The ninth topic underscores the rigorous procedures involved in nanocomposite synthesis and characterization. In contrast, the final topic touches on the intersection of magnetism with nanocomposites, hinting at applications in areas like medical imaging and data storage.

**Figure 10:**
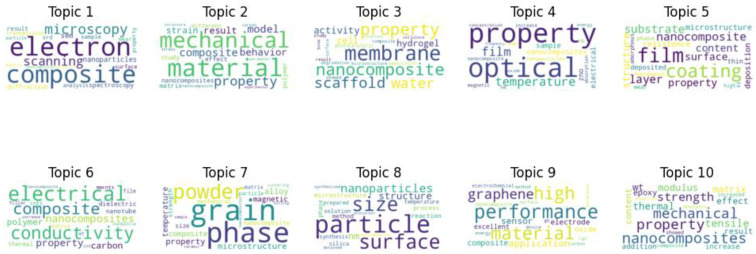
LDA-generated word clouds from abstracts.

Figure 10 provides illustrative word clouds for 10 distinct topics derived from scientific literature abstracts. The word size represents word prominence within each topic, offering quantitative insights into dominant terms for a comprehensive understanding of each topic’s relative importance.

Topic 1 primarily revolves around microscopy techniques for nanomaterial characterization. Terms like “electron”, “composite”, and “microscopy” suggest a focus on electron microscopy (EM) and advanced characterization methods like diffraction and spectroscopy. Topic 2 delves into nanocomposite mechanical properties, with terms like “mechanical”, “property”, and “strain” highlighting research on mechanical resilience and behavior. “Model” and “study” indicate theoretical and empirical frameworks. Topic 3 centers on biomedical applications, featuring words like “membrane”, “scaffold”, “hydrogel”, and “cell”. “Photocatalytic” hints at light-induced catalysis for therapeutic or environmental use. Topic 4 explores optoelectronic aspects, with “optical”, “temperature”, and “electrical”, suggesting investigations into thermal, electrical, and optical properties, often involving materials like zinc oxide (ZnO). Topic 5 focuses on thin-film technologies, emphasizing “film” and “coating”. Research may involve deposition techniques, substrate exploration, and surface morphology studies. Topic 6 revolves around electrical conductivity, with “carbon” and “polymer” indicating research on polymer-based nanocomposites reinforced with carbon fillers, potentially enhancing conductivity. Topic 7 highlights structural properties, possibly in metallic or ceramic composites, featuring terms like “grain”, “phase”, “microstructure”, “alloy”, and “magnetic”. Topic 8 delves into nanoscale phenomena, with “particle” and “size” indicating studies on nanoparticle synthesis, size-dependent properties, and their influence. Topic 9 centers on high-performance materials, especially those incorporating graphene. “High”, “performance”, “material”, “sensor”, and “electrode” suggest efforts to enhance material properties and applications. Topic 10 emphasizes nanocomposite mechanical robustness, with “mechanical”, “strength”, “tensile”, and “modulus”, highlighting research into tensile strength and modulus enhancement.

These topics reflect the diverse and expanding interest in nanocomposites across various fields, including materials science, electronics, and biomedicine.

### 3.4. Analysis of MAP and NET Files

Analysis of MAP and NET files is a pivotal aspect of bibliometric network analysis, particularly within the scope of VOSviewer. This analysis unveils the intricate interplay between Total Link Strength (TLS) and the frequency of specific terms. This quantitative exploration delivers invaluable qualitative insights into the research landscape.

**Figure 11:**
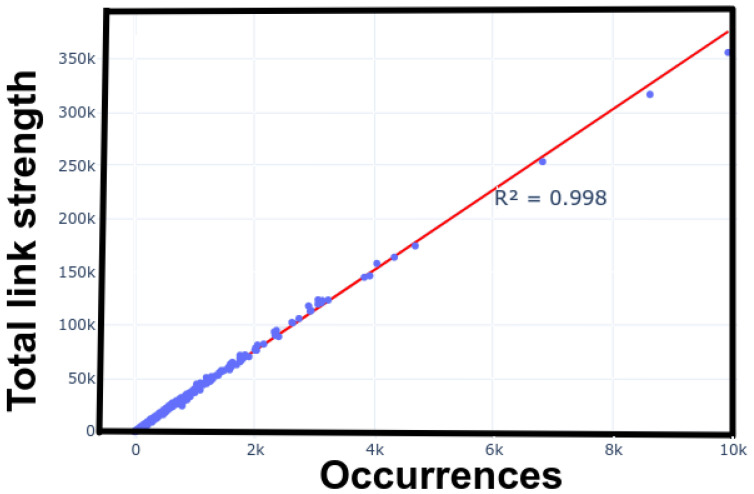
Total link strength vs. occurrences from VOS analysis.

TLS, in the context of VOSviewer, encapsulates an item’s connectivity and influence within a network, while occurrences quantify how often a term appears in the dataset. The relationship between these two metrics is elucidated in Figure 11, where each data point signifies a unique term’s position based on its occurrence frequency and cumulative link strength.

The most notable aspect of this analysis is the exceptionally high R^2^ value of 0.998, indicating a strong correlation. Essentially, as the occurrence of a term increases, its TLS proportionally increases. This finding suggests that terms with higher frequencies are not only popular but also centrally connected within the network, signifying their importance.

However, outliers in the scatter plot, characterized by high occurrences but relatively lower TLS, warrant scrutiny. These outliers may represent emerging or niche concepts that, despite frequent discussion, still need to integrate into the broader network of established terms.

Context plays a role in interpreting these data. Nascent research domains may experience rapid-term surges as foundational concepts are explored. As the domain matures, a more interconnected network of terms may emerge, resulting in a more uniform distribution of TLS and occurrences.

**Figure 12:**
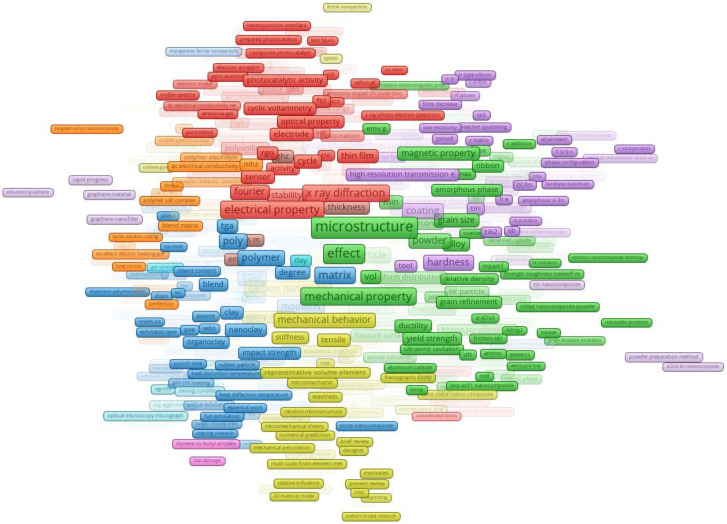
Network visualization from VOS analysis.

Figure 12 offers a detailed glimpse into the thematic clustering of terms in materials science, where clusters are formed based on shared terms, illuminating collaborative research trends within this field.

Cluster 1 revolves around surface characteristics, morphology, and electrical properties, reflecting a growing interest in electrical attributes of materials during 2016.3, possibly driven by advancements in electronic applications. Cluster 2 emphasizes the mechanical aspects of materials, particularly microstructure and mechanical properties. The focus on optimizing material strength and performance is evident, with a significant occurrence of “microstructure” in 2014.9. Cluster 3 highlights nanocomposites and their enhanced properties, with a surge in interest around 2015.6, possibly due to technological advancements enabling more sophisticated synthesis. Cluster 4 explores methodologies and specific nanomaterials, like carbon nanotubes, with “carbon nanotube” having 1911 mentions. These unique materials may hold promise for various industrial applications. Cluster 5 underscores the importance of characterization techniques, particularly electron microscopy, in understanding material structure, focusing on the mid-2010s. Cluster 6 delves into practical applications and their implications, with a more recent emphasis, possibly shifting from theoretical understanding to real-world use. Cluster 7 centers on nanoparticle integration into films, hinting at applications spanning electronics to coatings. Cluster 8 examines material properties, including resonant frequencies in technological applications, around 2016–2017. Cluster 9 focuses on membrane technologies, particularly improving water affinity properties for filtration and separation performances. Cluster 10 pertains to construction materials and their properties, aiming to enhance mechanical resilience during 2015–2017. Cluster 11 explores thermoelectric properties, focusing on materials that convert temperature differences into electric voltage, which is relevant for sustainable energy solutions. Cluster 12 encompasses various topics, including material properties of liquid solder alloys around 2016. Cluster 13 emphasizes advanced material characterization techniques, with a heightened focus during 2016–2019. Cluster 14 centers on innovative methodologies and their outcomes in material science, with a surge in novel approaches around 2015–2016. Cluster 15 delved into microscopic material characterization, mainly using scanning electron microscopy, around 2016.

In conclusion, Figure 12 unveils diverse research themes in materials science, reflecting evolving frontiers and the pursuit of knowledge and innovation within the scientific community.

**Figure 13:**
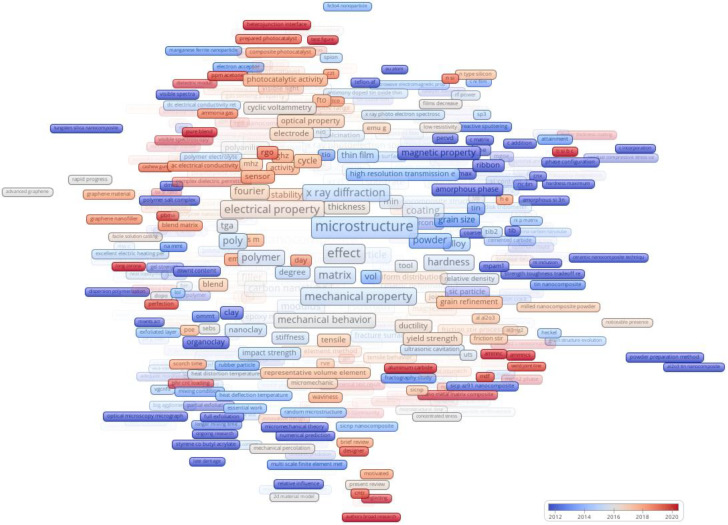
Overlay visualization from VOS analysis.

Figure 13 provides a comprehensive overview of recent terms within diverse clusters, offering insights into evolving trends in materials science.

Cluster 1 emphasizes optical properties and spectroscopy techniques, reflecting a growing interest in materials’ interaction with light, possibly due to advancements in optoelectronics. Cluster 2 focuses on hybrid and strengthened materials, emphasizing mechanical properties crucial for industries like aerospace and automotive. Cluster 3 highlights nanotechnology and material properties, with a surge in interest in carbon nanotubes and related properties. Cluster 4 delves into material durability and bonding properties, mentioning the specialized “Digimat software” for material modeling. Cluster 5 explores advanced fabrication and surface treatment techniques significant for applications like thin-film deposition and tribology. Cluster 6 intersects with biocompatible materials and their interactions, which are essential for medical applications. Cluster 7 relates to electronics and energy storage, highlighting novel material structures for energy devices or sensors. Cluster 8 signifies advancements in materials with heightened piezoelectric capacities relevant to renewable energy and energy-efficient designs. Cluster 9 centers on methodologies and material synthesis, focusing on membrane technology and moisture sorption for water purification.

Cluster 10 emphasizes nanotechnology challenges, particularly nanomaterial dispersion and composite material optimization. Cluster 11 underlines energy conservation and effective energy transition in materials, potentially in advanced ceramics or semiconductors. Cluster 12 suggests a broader acceptance of research methodologies or findings, referencing advanced materials. Cluster 13 showcases hybrid nanocomposites and materials with enhanced performance, reflecting evolving material science trends. Cluster 14 explores materials for extreme conditions relevant to nuclear or high-energy applications. Cluster 15 covers foundational research, highlighting methodological advancements and the relationship between material microstructure and performance. In conclusion, these clusters unveil a chronological narrative of research trajectories, reflecting the scientific community’s pursuit of knowledge, innovation, and technological advancements in materials science.

Acknowledging the complexity of the cluster figures due to the broad and varied groupings in the dataset, we have chosen the current presentation method as the most effective. Although a detailed analysis of each cluster is not the focus of this work, we aim to support the community’s needs. To this end, we have made the MAP and NET files publicly available on GitHub (https://github.com/ftir-mc/Nano-MEM, accessed on 25 October 2023), both the complete set and individual clusters, to facilitate more in-depth investigations by researchers. These files can be easily used with VOSviewer for further personalized analysis.

**Figure 14:**
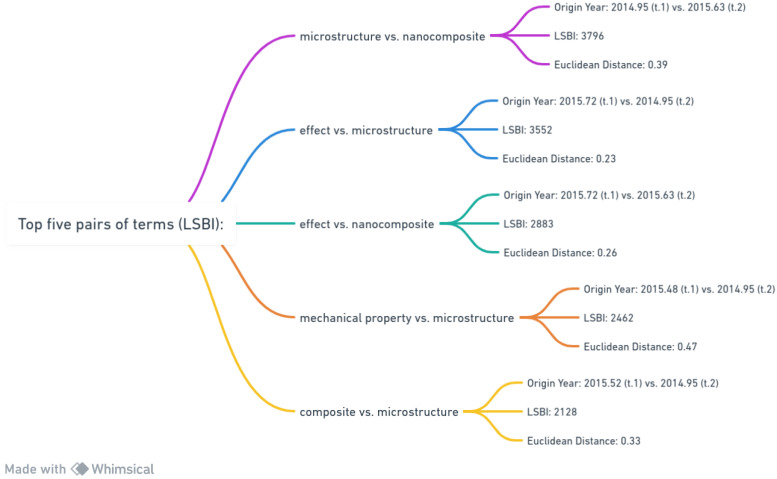
Top five pairs of terms depending on the value of LSBI.

Figure 14 offers insights into the top five pairs of terms based on their LSBI values, highlighting emerging relationships and research themes within a specific academic domain.

The pair “microstructure vs. nanocomposite”, originating in 2014.95 and 2015.63, boasts an LSBI of 3796 and a Euclidean distance of 0.39. This suggests a strong connection between these terms, indicative of a growing interest in understanding how nanocomposites and microstructures interact. Next, “effect vs. microstructure”, with origins in 2015.72 and 2014.95, exhibits an LSBI of 3552 and a Euclidean distance of 0.23. This pairing reflects a significant link between studying effects and microstructures during this timeframe. The “effect vs. nanocomposite” pair, emerging in 2015.72 and 2015.63, has an LSBI of 2883 and a Euclidean distance of 0.26, indicating a focus on the impact of effects on nanocomposites. The fourth pair, “mechanical property vs. microstructure”, from 2015.48 and 2014.95, has an LSBI of 2462 and a Euclidean distance of 0.47. This pairing suggests research efforts in correlating microstructures with mechanical properties. Lastly, “composite vs. microstructure”, originating in 2015.52 and 2014.95, carries an LSBI of 2128 and a Euclidean distance of 0.33, reflecting academic exploration into the relationships between composites and microstructures. In summary, Figure 14 unveils the complexities and research directions within a specific context, shedding light on scholarly pursuits related to effects, properties, and compositions concerning microstructures and nanocomposites. This portrayal emphasizes evolving research paradigms and invites further exploration of contextual factors and industry demands that may have influenced these academic trajectories.

**Figure 15:**
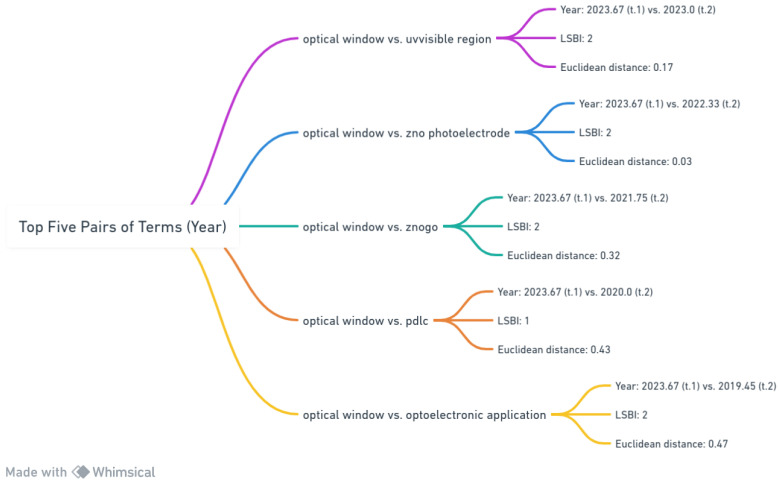
Top five pairs of terms depending on the value of Year.

Figure 15 provides an in-depth exploration of term relationships within optics and materials science. These data are distilled into five significant term pairs, revealing temporal dynamics and topological closeness using the Euclidean distance and Link Strength Between Items (LSBI).

The first pair, “optical window vs. uv visible region”, emerged in 2023.67 and 2023.0, indicating a growing focus on light permeability, possibly within the ultraviolet and visible spectrum. With an LSBI of 2, these terms are firmly linked, and the minimal Euclidean distance of 0.17 suggests subtle contextual variations or closely related subdomains. This proximity may signify an emerging trend or innovation in this specific domain during that period. The next pair, “optical window vs. zno photoelectrode”, originating in 2023.67 and 2022.33, reflects advancing research on zinc oxide-based photoelectrodes, likely in photovoltaic or photoelectrochemical cells, and their optical properties. The LSBI of 2 and a minimal Euclidean distance of 0.03 indicate near-synonymous usage, highlighting significant overlap in associated research areas. The third pairing, “optical window vs. znogo”, with origins in 2023.67 and 2021.75, suggests exploring optical characteristics related to the “znogo” compound or material. The LSBI is similar to the previous pair, but the increased Euclidean distance of 0.32 suggests a broader contextual separation, potentially indicating diverse applications or expanding research scope. In the fourth pairing, “optical window vs. pdlc”, a more significant temporal gap is observed from 2023.67 to 2020. This may indicate a resurgence of the Polymeric Liquid Crystal Display (PDLC) technology and its optical attributes. An LSBI of 1 suggests a moderate correlation, while a substantial Euclidean distance of 0.43 implies distinctions in applications or research objectives. Finally, “optical window vs. optoelectronic application”, originating in 2023.67 and 2019.45, demonstrates the widest temporal difference among the pairs. This pairing likely explores various optoelectronic applications of optical windows, enhancing electronic or photonic device performance. The significant Euclidean distance of 0.47, combined with an LSBI of 2, suggests a deep-rooted yet expansive connection. In summary, Figure 15 unveils the intricate relationships among pivotal terms in optics and materials science. These relationships, considering both temporal emergence and interconnectedness, offer valuable insights into evolving research trajectories and potential technological innovations in the field. This analysis emphasizes the importance of monitoring these dynamics to foster a comprehensive understanding of the field’s progress.

### 3.5. Bibliometric Data Analysis and Visualization Report

**Figure 16:**
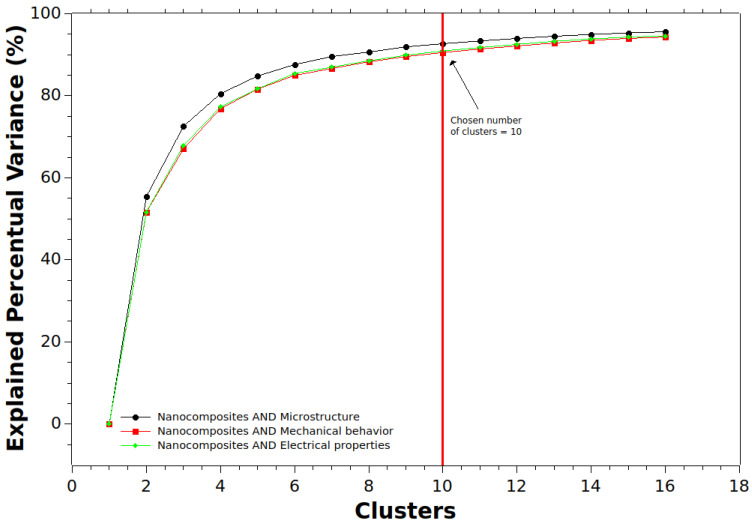
Elbow plot.

Figure 16 presents a concise visualization of the optimal cluster number determination using the Elbow Method across three vital datasets: “nanocomposites and microstructure”, “nanocomposites and mechanical behavior”, and “nanocomposites and electrical properties”. This method aids in pinpointing the ideal number of clusters, signifying where additional clusters cease to reduce percentage variance significantly.

For the “nanocomposites and mechanical behavior” dataset, the Elbow Method reveals that a 10-cluster model effectively captures around 90.53% of the explained variance. Beyond this point, the model’s efficiency plateaus, indicating that 10 clusters represent the underlying structure in the mechanical behavior data.

Similarly, “nanocomposites and microstructure” analysis benefits from a 10-cluster configuration, explaining an impressive 92.69% variance. The clear transition from a steep incline to a gradual progression underlines the significance of the 10-cluster configuration in encapsulating microstructural aspects effectively.

In the domain of “nanocomposites and electrical properties”, a parallel trend emerges, with a 10-cluster arrangement capturing approximately 90.92% of the explained variance. The trajectory of this dataset mirrors the prior two, reinforcing the diminishing returns beyond the 10-cluster threshold.

These outcomes prompt contemplation of potential contextual factors. The intricate nature of nanocomposites, involving multiple compositional elements and properties, may lead to shared behavioral patterns. This convergence could result from shared research methodologies, everyday materials, or shared challenges.

Furthermore, bibliometric data nuances, such as frequently researched themes or prevailing trends, may lead to keyword concentration around specific topics. This could explain the consistent emergence of the 10-cluster model across the three datasets.

#### 3.5.1. Nanocomposites and Electrical Properties

**Figure 17:**
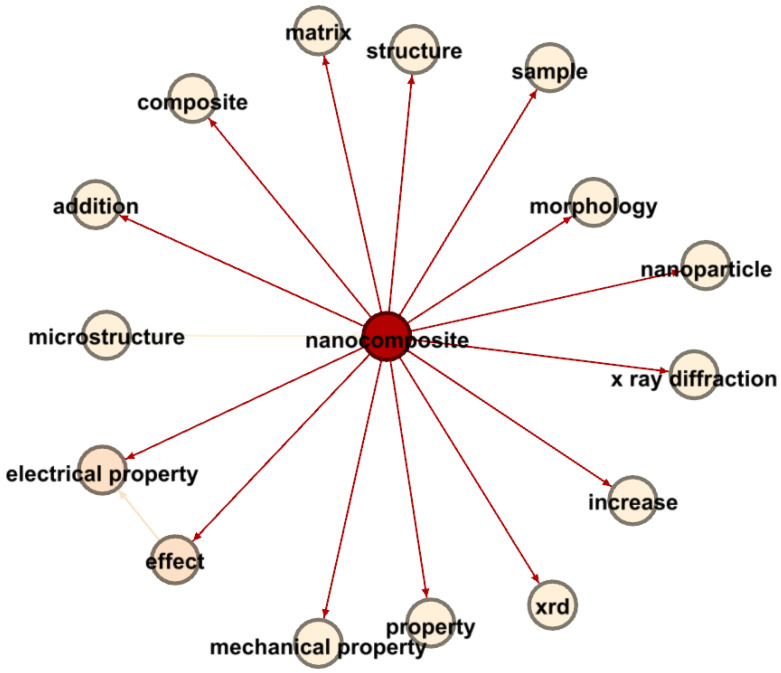
Network illustrating the strength of links between terms related to nanocomposites and electrical properties.

In the realm of nanocomposites and electrical properties, Figure 17 provides a complex visual representation that highlights the relationships between research terms, primarily through Link Strength Between Items (LSBI) values and average publication years. The central term, “nanocomposite”, is a crucial node connecting various related terminologies.

Significant findings include the strong association between “microstructure” and “nanocomposite”, with an LSBI of 3796 and an average publication year of 2015.29, indicating a profound interest in understanding how microstructural attributes influence nanocomposite properties during that period.

Moreover, the connection between “nanocomposite” and “electrical property”, characterized by an LSBI of 1959 and an average publication year of 2015.955, reveals a recent research focus on the electrical aspects of nanocomposites. The link with “effect”, having an LSBI of 2883 and an average publication year of 2015.68, indicates extensive investigations into the influence of nanocomposites on various properties, including electrical characteristics.

Additionally, the connection between “effect” and “electrical property”, with an LSBI of 1288 and an average publication year of 2016, emphasizes the growing attention given to discerning the specific effects of nanocomposites on electrical properties.

**Figure 18:**
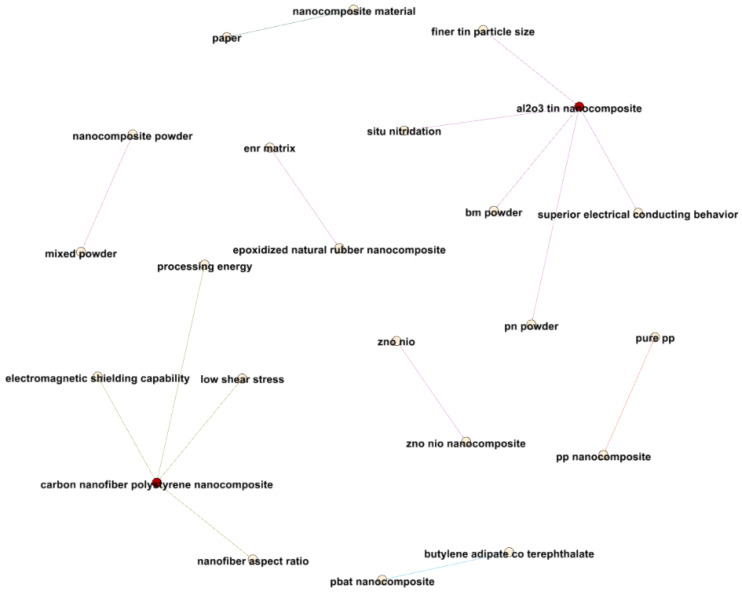
Network of closest terms related to nanocomposites and electrical properties.

Figure 18 delves into closer relationships between terms related to nanocomposites and electrical properties, revealing valuable insights. For instance, a strong connection between “nanocomposite material” and “paper”, with an average year of 2014.565, suggests pivotal research in 2014 concerning nanocomposite materials.

The relationship between “pp nanocomposite” and “pure pp”, with an average year of 2014.625, underscores the heightened research focus during this period on distinguishing between PP nanocomposites and pure PP.

Connections between various nanocomposite formulations and their associated properties, ranging from 2011 to 2020, illustrate the evolving nature of nanocomposite research. For instance, the “zno nio” and “zno nio nanocomposite” terms in 2020 signify expanded research into the broader applications of these nanocomposites.

**Figure 19:**
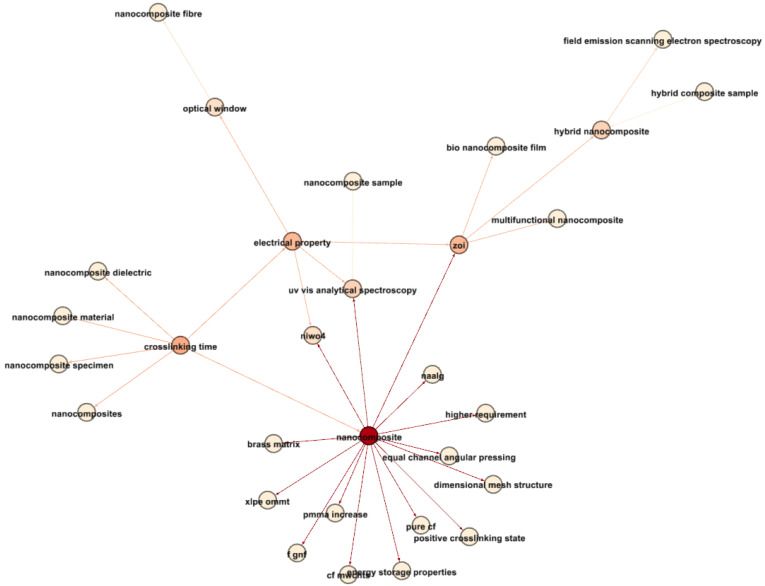
Network illustrating the newest terms related to nanocomposites and electrical properties.

Figure 19 concentrates on the newest terms related to nanocomposites and electrical properties. Notably, “crosslinking time” emerges as a critical parameter influencing nanocomposites. The connection between “crosslinking time” and “nanocomposite material” suggests significant research around 2018, highlighting the critical role of crosslinking in nanocomposite investigations.

Furthermore, nanocomposites exhibit strong associations with “energy storage properties”, “equal channel angular pressing”, and “dimensional mesh structure”, reflecting the multifaceted nature of recent nanocomposite studies.

Additionally, links between nanocomposites and “optical window” and “uv-vis analytical spectroscopy” signify research into the electrical–optical behaviors of nanocomposites, potentially within optoelectronic devices.

In summary, this analysis illuminates key trends in nanocomposites and electrical properties research. Recent studies emphasize understanding the relationships between nanocomposite materials and their electrical attributes. Crosslinking time plays a pivotal role in shaping nanocomposite properties. Explorations into energy storage and electrical–optical behaviors represent significant areas of investigation. Collaborations among experts in materials science, electrical engineering, and analytical techniques can drive innovations in nanocomposite research, advancing the field into new frontiers.

#### 3.5.2. Nanocomposites and Mechanical Behavior

**Figure 20:**
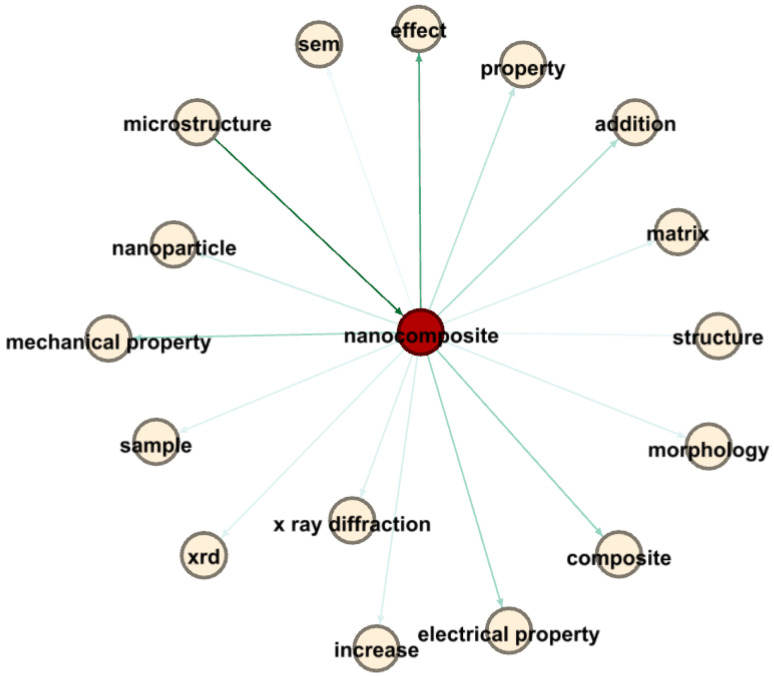
Network illustrating the strength of links between terms related to nanocomposites and mechanical behavior.

In nanocomposites and mechanical behavior, Figure 20 offers a comprehensive view of the connections between research terms, primarily through Link Strength Between Items (LSBI) values. Central to this analysis is the term “nanocomposite”, a hub for various related terminologies.

Key findings include a significant link between “microstructure” and “nanocomposite”, marked by a substantial LSBI value of 3796. This connection underscores the importance of microstructural details in shaping nanocomposites’ properties. The average publication year for this connection centers around 2015, indicating sustained interest during that period.

Furthermore, “effect” plays a crucial role in nanocomposite research, with an LSBI value of 2883 for the link between “effect” and “nanocomposite”. This signifies extensive exploration into the consequences of incorporating nanoparticles into different matrices.

The focus extends to nanocomposites’ properties, encompassing both “mechanical” and “electrical” aspects, as indicated by the robust association between “nanocomposite” and “mechanical property”, characterized by an LSBI of 1941.

Terms like “addition” and “increase” also hold importance, with LSBI values of 1801, suggesting the incorporation of various substances or nanoparticles to enhance base material properties.

The role of analytical techniques is prominent, as terms like “x-ray diffraction”, “XRD”, and “sem” underscore the significance of advanced analytical methods in understanding nanocomposite structure and morphology.

Interdisciplinary connections are abundant, with terms like “composite”, “matrix”, “morphology”, “sample”, and “structure” having varying LSBI values, illustrating the comprehensive research framework in which nanocomposites are studied.

In essence, the network of connections revolving around “nanocomposite” provides a vivid depiction of the extensive research landscape it encompasses. This narrative highlights nanocomposites’ pivotal role in modern materials science, particularly their mechanical behavior. Researchers take a multifaceted approach to unravel the complexities of nanocomposites, leading to innovations in various applications.

**Figure 21:**
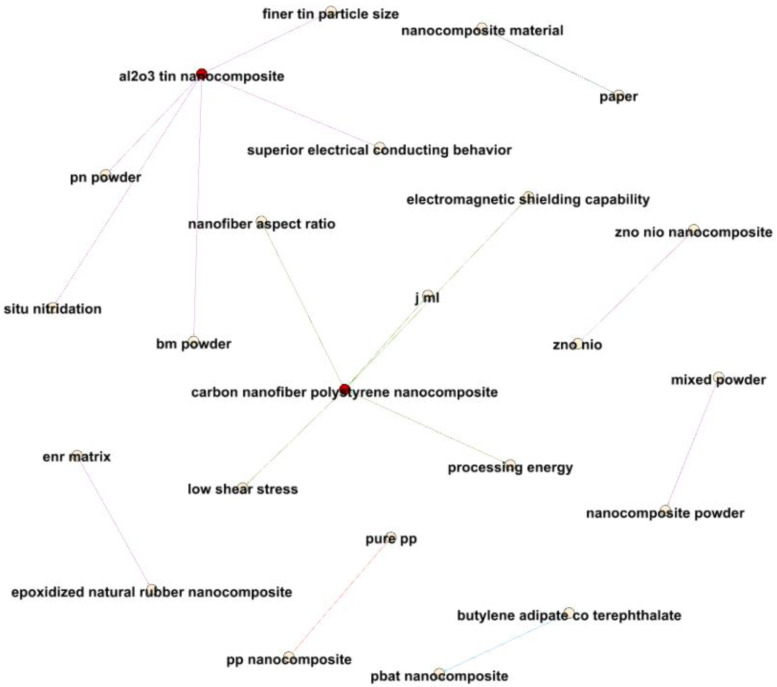
Network of closest terms related to nanocomposites and mechanical behavior.

Figure 21 delves into closer relationships between terms related to nanocomposites and mechanical behavior, revealing significant insights. For example, a robust connection exists between “nanocomposite material” and “paper”, indicating substantial academic publishing in nanocomposites, emphasizing around 2014.

The data analysis reveals significant findings in nanocomposites and their mechanical behavior. The initial dataset highlights a strong connection between “nanocomposite material” and “paper”, indicating substantial academic research, particularly around 2014.

Another noteworthy observation is the relationship between “pp nanocomposite” and “pure pp”, indicating the study of polypropylene (PP)-based nanocomposites derived from pure PP. This relationship, although modest with an LSBI value of 7, points to extensive documentation in the literature.

The emergence of PBAT nanocomposites becomes evident through the connection between “butylene adipate co terephthalate” (PBAT) and “pbat nanocomposite”, signifying the evolution of PBAT-based nanocomposites, particularly around 2018.

A complex network segment surrounds “Al_2_O_3_ tin nanocomposite”, linking to various nodes such as “finer tin particle size”, “superior electrical conducting behavior”, “in-situ nitridation”, and various powders. This suggests a thorough exploration of the properties, synthesis methods, and applications of alumina–tin nanocomposites, primarily around 2009.

The term “carbon nanofiber polystyrene nanocomposite” connects to concepts like “electromagnetic shielding capability”, “nanofiber aspect ratio”, and “processing energy”, implying investigations into its mechanical attributes, processing methodologies, and potential applications, predominantly around 2013.

Lastly, “zno nio nanocomposite” is linked to its base components, “zno nio”, indicating recent research into zinc oxide–nickel oxide nanocomposites, notably in 2020.

**Figure 22:**
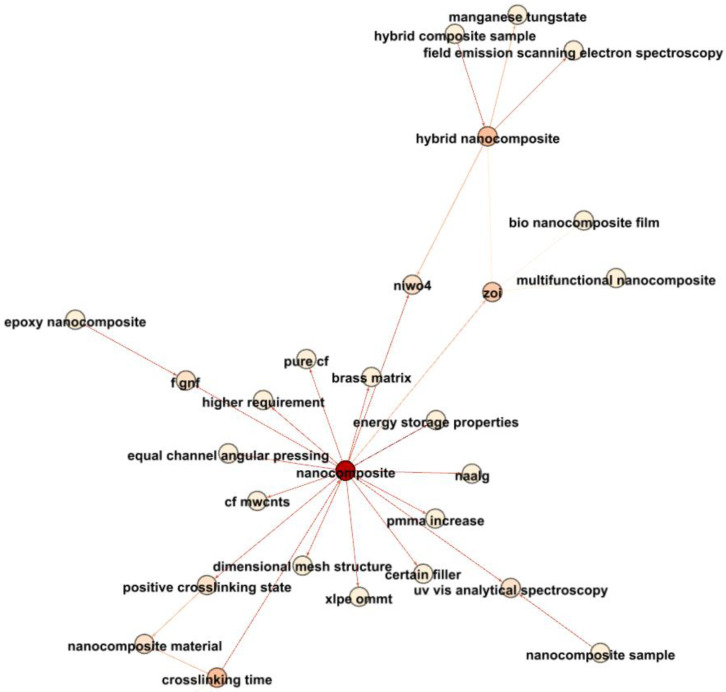
Network illustrating the newest terms related to nanocomposites and mechanical behavior.

Figure 22 visually represents the relationships between various terminologies in nanocomposites and their mechanical behavior, focusing on the latest terms. It provides insights into evolving research trends, collaborations, literature gaps, and patterns, concentrating on terms like “nanocomp.*” and “.*echanical behav.*”.

The data emphasize the significance of crosslinking time in nanocomposite research, particularly toward the end of the second decade of the 21st century. The robust connection between crosslinking time and the general term “nanocomposite” suggests recurrent exploration of how crosslinking durations impact nanocomposite properties, with an average year around 2019.44.

There is a notable correlation between nanocomposites and their energy storage properties, indicating potential advancements in energy storage solutions, possibly in advanced batteries or supercapacitors. Simultaneously, there is a trend toward multifunctional nanocomposites, emphasizing materials that offer multiple benefits, optimizing performance and cost.

The relationship between nanocomposites and terms like “equal channel angular pressing”, “dimensional mesh structure”, and “brass matrix” suggests a deep dive into the mechanical processes involved in nanocomposite formulation. Additionally, advanced analytical techniques such as “field emission scanning electron spectroscopy” are highlighted, underscoring the need for high-resolution structural and morphological analysis.

Emerging trends point toward integrating nanocomposites into specialized domains. For instance, the connection between “nanocomposite fiber” and “optical window” suggests potential applications in optoelectronics. Terms like “zoi” (Zone of inhibition) bridge the gap between hybrid nanocomposites and bio-nanocomposite films, hinting at possible biocompatible or bio-derived nanocomposite materials.

In summary, these data unveil the dynamic landscape of nanocomposite research, progressing from fundamental studies in the early 2010s to more specialized investigations. The field demonstrates relentless innovation, focusing on crosslinking time, energy storage, multifunctionality, and specialized applications. Researchers are encouraged to address gaps in optimizing crosslinking times, explore multifunctional nanocomposites, and integrate nanocomposites into various applications. The potential of nanocomposites in shaping a sustainable technological future is undeniable, and continued research in this domain is essential.

#### 3.5.3. Nanocomposites and Microstructure

**Figure 23:**
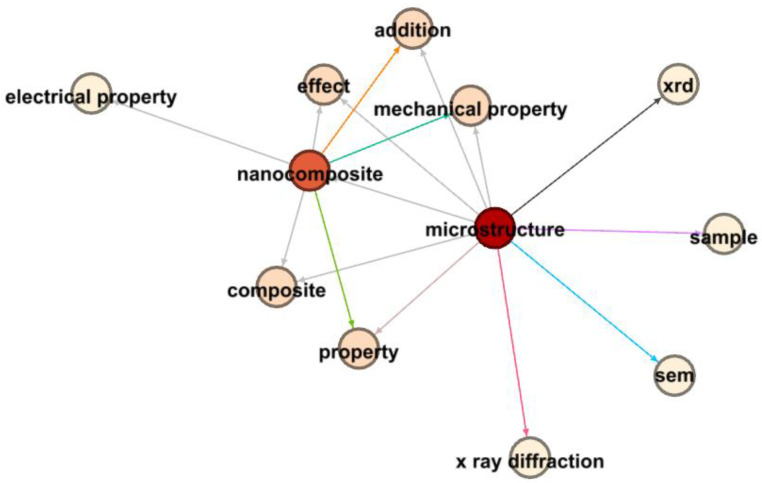
Network illustrating term associations in nanocomposites and microstructure.

In the context of nanocomposites and their mechanical properties, a presented bibliometric study, depicted in Figure 23, provides a comprehensive overview of the intricate relationships among various research terms and their associated parameters. At the core of this analysis lies the Link Strength Between Items or Terms (LSBI), offering a quantitative measure of these associations.

Within this intricate network of research concepts, the study uncovers numerous relationships, shedding light on the interconnected nature of terms in the field. The central focus of this examination revolves around “microstructure” and “nanocomposite”, which exhibit a wide array of connections to various thematic areas, emphasizing their pivotal roles in multidisciplinary research.

The initial dataset highlights a robust connection between “microstructure” and “nanocomposite”, characterized by an LSBI value of 3796 and a DE of 0.39. This strong association signifies a profound correlation between the detailed arrangement of internal constituents (microstructure) and materials enhanced with nanoscale reinforcements (nanocomposites). The average year of this correlation, approximately 2015.29, suggests that this relationship has gained notable significance in recent years, marking a surge in understanding how microstructural characteristics influence nanocomposite properties and performance.

Further exploration of “microstructure” reveals diverse connections, including a robust link with “effect” (LSBI: 3552), indicating substantial research into how microstructures influence or are influenced by various external and internal factors. Additionally, a strong connection with “mechanical property” (LSBI: 2462) underscores the pivotal role of microstructure in determining material mechanical behavior. “Composite”, with an LSBI of 2128, delineates the fundamental interplay between material microstructures and composite materials. Moreover, connections with “addition”, “property”, “x-ray diffraction”, “XRD”, “SEM”, and “sample” represent the comprehensive scope of microstructural studies, from material property evaluations to intricate analysis techniques.

As we shift our focus to “nanocomposite”, its dynamic interactions with various terms mirror its versatility. For instance, the connection with “effect” (LSBI: 2883) echoes studies on the impact of integrating nano-reinforcements into composites. “Composite” emerges as a direct relation with an LSBI of 2066, highlighting the foundational connection between conventional composites and their nanoscale counterparts. A link with “electrical property” (LSBI: 1959) offers insight into the exploration of nanocomposites in electronic and electrical applications. Other prominent connections encompass “mechanical property”, “addition”, and “property”, revealing the multifaceted nature of nanocomposite research.

These intricate relationships between “microstructure” and “nanocomposite”, along with their associated nodes, underscore the complex interdependencies within the research domain. These strong interconnections hint at an integrated approach where understanding the micro-level intricacies of materials profoundly shapes the properties and applications of advanced nanocomposites at the nano level.

The data paint a vibrant picture of a scientific domain characterized by interconnectedness and continuous evolution. One plausible scenario entails the development of advanced nanocomposites tailored for specific applications, driven by a deep understanding of their microstructure. This could lead to innovations in sectors ranging from aerospace to electronics, where material performance is paramount.

**Figure 24:**
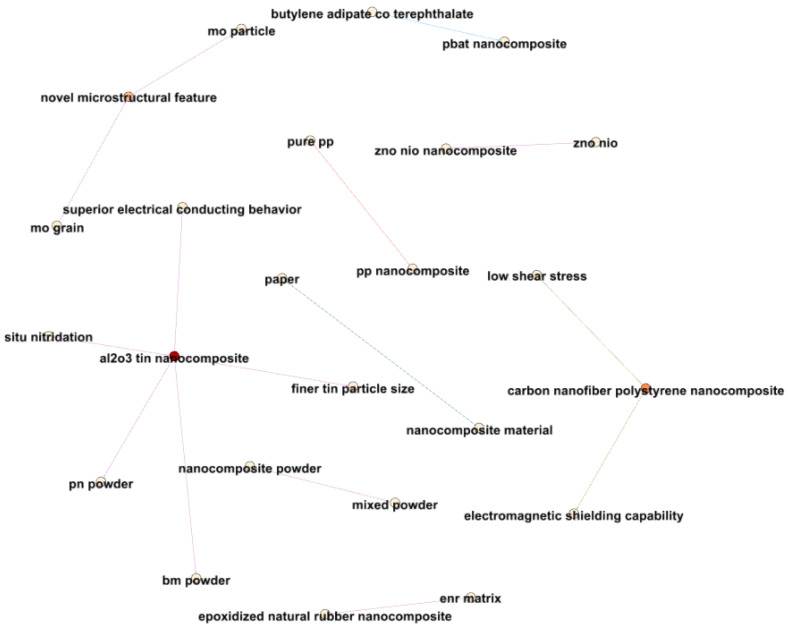
Network of closest terms related to nanocomposites and microstructure.

Figure 24 illustrates relationships among closely related terms in nanocomposites and microstructure, employing the Euclidean distance metric to reveal intricate interactions primarily centered on nanocomposites and their microstructure.

In the expansive realm of scientific research, network graphs, like the one presented, serve as potent tools to uncover subtle relationships and trends within complex datasets. In this exploration of bibliometric data, the primary focus is on nanocomposites and microstructural features.

Nanocomposite Material and its Relations: The initial dataset draws attention to the association between “nanocomposite material” and “paper”. With an average year value of 2014.565 and a substantial LSBI value of 49, it is clear that research papers and publications discussing nanocomposite materials have notably increased during this period. This mirrors the global interest in nanocomposites due to their enhanced mechanical, thermal, and electrical properties.

A noteworthy link surfaces between “pp nanocomposite” and “pure pp”, with an average year value of 2014.625. Polypropylene (PP) nanocomposites have gained attention for their improved mechanical strength and barrier properties compared to pure PP counterparts. The LSBI value of 7 suggests a moderate connection, indicating ongoing research in modifying polypropylene with nanomaterials.

Delving into Microstructural Themes: Notable connections from around 1995 involve “mo grain”, “novel microstructural feature”, and “mo particle”. These connections underscore a specific interest in the microstructural attributes of molybdenum (Mo) during this period, shedding light on material behavior through microstructural analysis.

Evolution of Nanocomposite Research: The connection between “butylene adipate co terephthalate” and “PBAT nanocomposite”, with an average year value of 2018.685, points to recent advancements in polymer blend nanocomposites. PBAT, a biodegradable copolyester, holds promise for sustainable and high-performance materials in nanocomposite form.

An intriguing cluster centered around “Al_2_O_3_ tin nanocomposite” in 2009 reveals connections to terms like “bm powder”, “finer tin particle size”, “pn powder”, “situ nitridation”, and “superior electrical conducting behavior”. This suggests intensive studies on the electrical and structural properties of Al_2_O_3_ tin-based nanocomposites during that time.

Emerging Themes in Nanocomposite Research: Notably, the link between “carbon nanofiber polystyrene nanocomposite” and associated properties and applications, such as “electromagnetic shielding capability”, points to 2013. This suggests that incorporating carbon nanofibers into polystyrene matrices may enhance the material’s ability to shield against electromagnetic interference, particularly significant in the electronics industry.

In summary, a nuanced examination of the connections in this bibliometric dataset reveals several patterns. The persistent evolution of nanocomposite research from the 1990s to today underscores its significance in material science. The shift from foundational studies on microstructural properties in the 1990s to application-driven research in electromagnetic shielding and sustainable materials in the 2010s reflects the field’s maturation.

**Figure 25:**
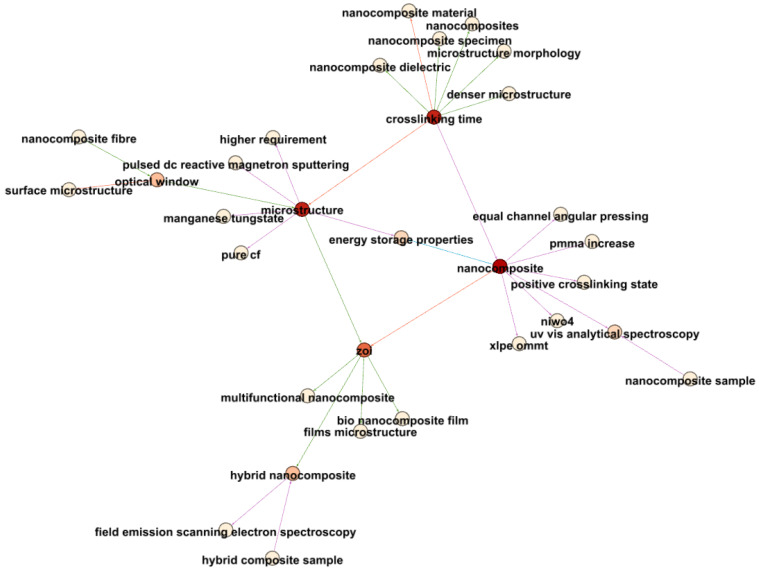
Network illustrating the newest terms related to nanocomposites and microstructure.

Figure 25 presents a bibliometric network graph, showcasing intricate relationships among various nanocomposite and microstructure terms, focusing on the newest terms. This analysis reveals evolving research trends, collaborations, and emerging patterns.

In nanocomposite research, this bibliometric analysis delves into the intricate associations between various nanocomposite-related terms, focusing on their mechanical properties. The study employs network graph methodologies to elucidate these connections, revealing the term “crosslinking time” as a nexus point that frequently co-occurs with “nanocomposite”, “microstructure”, and “nanocomposite material”. This indicates a concentration of scholarly attention between 2018 and 2020.

The linkage between “optical window” and “surface microstructure”, with an average mention year close to 2020, signals an active area of research that probes into nanocomposites’ visual and surface characteristics. Moreover, “energy storage properties” emerge prominently within the literature, often in conjunction with “microstructure” and “nanocomposite”, which underscores the increasing significance of nanocomposites within the energy storage sector.

The central role of “microstructure” in the performance and application of nanocomposites is underscored by its connection to terms such as “manganese tungstate” and “higher requirement”. This association underscores the critical importance of microstructural integrity in enhancing nanocomposite functionality.

Further analysis reveals that terms like “nanocomposite fiber” and “optical window” are being researched in light transmission through nanostructured fibers. The term “zoi”, or zone of inhibition, presents an intriguing connection to both “microstructure” and “nanocomposite”, suggesting a nascent property or technique emerging in nanocomposite research.

The pairing of “UV-Vis analytical spectroscopy” with “nanocomposite” reflects recent advancements in employing spectroscopic methods for analyzing these materials, with a notable focus on studies around 2019.

References to “pure CF” about “microstructure” point to an interest in the microstructural investigation of pure carbon fibers in contemporary studies.

This bibliometric investigation presents an overarching perspective on nanocomposites, singling out “crosslinking time” and microstructural integrity as crucial determinants of their mechanical behavior. There is also an evident surge in interest in their optical qualities and capabilities in energy storage, with implications for optoelectronics and sustainable energy technologies.

Novel terms such as “zoi”, “NiWO_4_”, and “UV-Vis analytical spectroscopy” surface within the study, hinting at specialized subfields that could be at the cusp of innovative developments. As the comprehension of nanocomposites and their microstructural characteristics deepens, one can anticipate the advent of advanced materials designed with precise functionalities, which could revolutionize various industrial sectors, from electronics to aerospace.

The anticipation is that cross-disciplinary collaboration among optoelectronics, mechanical engineering, and materials science experts will propel advancements in sustainable energy storage, advanced protective coatings, and the next wave of electronic devices. Specialized terminologies and methodologies are expected to facilitate exacting control over material properties, culminating in the creation of custom-tailored nanocomposites for niche applications.

The application of data mining and machine learning methodologies in this study shines a light on the multifaceted landscape of nanocomposites, prioritizing the nuanced characterization of microstructure, electrical properties, and mechanical behavior. This approach reveals the dominant research trajectories and the complex interrelationships within the various dimensions that define nanocomposite materials.

Exploring the Scopus dataset reveals a significant evolution in global research dynamics, with contributions from diverse nations, democratizing nanocomposite research. Collaboration is evident through joint publications, emphasizing the integrative and interdisciplinary nature of current research.

Textual analytics track the evolution of terminologies, emphasizing emergent terms related to mechanical behavior, such as ZnO-NiO nanocomposites. Bibliometric and network graph methodologies provide insights into keyword interrelationships, enhancing understanding of temporal trends and dynamic research foci in the nanocomposite field.

In summary, this study blends computational methodologies with traditional nanomaterial research to deepen our data-driven understanding of nanocomposites, laying a robust foundation for future endeavors in this rapidly evolving domain.

## 4. State of the Art and Gaps Extracted from Results and Discussions

Section 4 delves into the contemporary landscape of nanocomposite research as of 23 October 2023. Drawing from diverse sources and methodologies, including Scopus database analysis, textual analytics, and bibliometric data, this segment meticulously charts the expansive terrain of nanocomposites. It highlights critical research themes, historical trajectories, and emerging trends, focusing on microstructure, electrical characteristics, mechanical dynamics, and nanocomposite films. Collaboration is crucial for innovation, and highly cited articles offer a window into the field’s pulse. The emergence of new research avenues, China’s significant financial contributions, and linguistic shifts signify the evolving dynamics of this domain. As Section 4 concludes, it not only furnishes a holistic overview of current nanocomposites research but also illuminates the path forward for future scholarly endeavors.

### 4.1. Insights from Section 3.1: Scopus Database Search Strategy

Analysis of the Scopus Database Search Strategy reveals a vast nanocomposite research landscape comprising 209,012 documents as of 23 October 2023. This research prominently explores microstructural investigations, delving into the microscopic arrangement of materials and its impact on properties. While research on electrical properties is less voluminous, it plays a vital role in understanding these materials. Mechanical behavior remains a significant study area, especially regarding material responses under various stress conditions.

Historical trends show that nanocomposite research gained prominence in the late 1970s, peaking between 2018 and 2022, reflecting a shift toward understanding nanocomposite attributes, exemplified by the term “properties”. Research on nanocomposite films has also grown, finding applications in coatings and barriers. The role of tools like scanning electron microscopy (SEM) in elucidating nanocomposite microstructures is underscored.

However, post-2022, there is a noticeable decline in nanocomposite research, potentially indicating research saturation, evolving priorities, or emerging challenges. Reduced emphasis on “increase” may suggest limited investigations into scalability and broader acceptance. The increased focus on “effect” may signal growing interest in outcomes and potentialities, requiring further exploration.

In conclusion, key research areas for future exploration encompass microstructure in nanocomposites, electrical properties of nanocomposites, mechanical behavior of nanocomposites, nanocomposite films, and SEM in nanocomposite research. This comprehensive analysis offers valuable insights into the current state of nanocomposites research and identifies potential directions for future scholarly endeavors.

### 4.2. Insights from Section 3.2: Textual Analytics Approach

Collaborations prove pivotal for innovation, with prominent scholars like Sekino, Tohru, and Niihara, Koichi, along with contributions from researchers like Chang, W.C., highlighting the potency of collaborative research in nanocomposites.

Highly cited articles, such as “High-thermoelectric performance of nanostructured bismuth antimony telluride bulk alloys”, indicate influential studies. Investigating the reasons behind their citation surge can reveal research inclinations and knowledge gaps in nanocomposites.

Emerging trends focus on “Hierarchically porous Co/C nanocomposites for ultralight high-performance microwave absorption” and “High-strength Damascus steel by additive manufacturing”. These signal new research avenues in electromagnetic interference shielding, sustainable materials, and bioinspired designs.

China’s increasing funding in nanocomposite studies raises questions about its motivations and consequences. Understanding China’s investment rationale can provide insights into the nanocomposite research trajectory.

The rise of the Chinese language in nanocomposite studies suggests an area of exploration regarding linguistic diversity’s impact on knowledge dissemination and collaboration.

Sentiment analysis of academic articles reveals growing optimism in nanocomposite research, offering insights into the research ambiance and areas of enthusiasm and concern.

To explore 2023 trends in nanocomposite research comprehensively, recommended keywords include (i) Nanocomposite Collaborations, (ii) Highly Cited Nanocomposite Articles, (iii) Emerging Trends in Nanocomposites, (iv) Global Funding in Nanocomposites, focusing on China’s role, (v) Language Trends in Nanocomposite Research, and (vi) Sentiment Analysis in Nanocomposite Abstracts. Utilizing these keywords empowers readers with the latest insights in the dynamic field of nanocomposite research.

### 4.3. Insights from Section 3.3: Scholarly Literature Analysis on Nanocomposite Themes

The scholarly literature analysis on nanocomposite themes reveals the intricate and multifaceted nature of research in this field. Nanocomposite research covers many themes, including analysis, synthesis, performance enhancement, and more. Notably, advanced characterization techniques like electron microscopy, diffraction, and spectroscopy play a significant role, reflecting the continuous evolution of tools and methods for comprehensive nanocomposite analysis.

The focus on understanding the mechanical behavior of nanocomposites is evident, bridging gaps in performance and application knowledge. Biomedical nanocomposite applications are also rising, suggesting potential roles in tissue engineering and regenerative medicine.

Optoelectronic properties, particularly in materials like zinc oxide, are a growing area of interest due to their applications in optoelectronics. Ongoing research into thin-film technologies sheds light on understanding nanocomposite film properties and applications. Electrical conductivity, especially in carbon-based filler-reinforced nanocomposites, is a significant domain, highlighting their importance in electronics.

Continued emphasis on structural properties and nanoparticle phenomena underscores the need to understand these materials’ intrinsic characteristics. High-performance materials, particularly those incorporating graphene, signify efforts to enhance material properties for applications ranging from sensors to electrodes.

To stay updated on the 2023 trends in nanocomposite research, recommended keywords include (i) Nanocomposite Research Themes, (ii) Advanced Characterization Techniques, (iii) Biomedical Nanocomposites, (iv) Optoelectronic Nanocomposites, (v) Thin-Film Nanocomposites, (vi) Conductive Nanocomposites, (vii) Structural Analysis of Nanocomposites, (viii) Nanoparticle-Enhanced Materials, and (ix) High-Performance Nanocomposites, with a focus on graphene-based ones. These keywords empower researchers to access the latest articles and data, ensuring a comprehensive and updated view of the dynamic nanocomposite research field in 2023.

### 4.4. Findings from Section 3.4: Analysis of MAP and NET Files

The analysis of MAP and NET files, using VOSviewer, provides valuable insights into bibliometric network analysis, extending beyond the specific domain to broader subjects like materials science and optics. Several essential findings and areas of investigation have emerged.

One significant discovery is a strong correlation, with an R^2^ value of 0.998, between term frequency and Total Link Strength (TLS). This indicates that frequently appearing terms hold central positions in the research landscape, solidifying their importance. However, certain outliers have been identified—terms that occur frequently but have lower TLS values. These outliers may represent emerging or specialized topics that have yet to fully integrate into the recognized network of terms, potentially indicating novel research directions.

It is crucial to consider the evolutionary stage of the research domain, as emerging areas may experience rapid-term spikes. In contrast, established ones exhibit more balanced distributions between TLS and term occurrences. Thematic clusters have also emerged, highlighting prominent research themes, including surface characteristics, mechanical properties, nanocomposites, and material characterization methods. Temporal trends, such as the increased focus on materials’ electrical properties around 2016, suggest growing interest in electronic applications.

In terms of gaps and opportunities, the outliers deserve special attention as potential subjects for further investigation, given their relevance and potential role in shaping new research directions. Monitoring the maturation and transformation of research domains can provide insights into the interplay between term occurrences and TLS over time, revealing overarching research patterns. Emerging themes within clusters, especially in areas like nanocomposites, warrant thorough examination. Understanding shifts in research focus over time, such as the 2016 shift toward electrical properties, can offer insights into influential factors driving research directions. Examining the relationships among terms within clusters, particularly recent term combinations with notable LSBI values, can signal the emergence of new research themes.

To stay current with the 2023 trends in bibliometric network analysis, recommended keywords include (i) Bibliometric Network Analysis Themes, (ii) Total Link Strength (TLS), (iii) Occurrences, (iv) Outliers in Bibliometric Analysis, (v) Evolutionary Stage of Research Domains, (vi) Thematic Clustering in Materials Science, (vii) Temporal Trends in Research Topics, (viii) Emerging Research Trends, (ix) Interconnectedness of Terms, and (x) Research Themes in Optics and Materials Science. These keywords will facilitate a comprehensive search for relevant articles and studies, enhancing understanding of bibliometric network analysis and related research domains.

### 4.5. Findings from Section 3.5: Bibliometric Data Analysis and Visualization Report

The analysis in Section 3.5, focused on bibliometric data analysis and visualization, provides valuable insights into the nanocomposite research landscape. Key findings are summarized in three subsections.

#### 4.5.1. Nanocomposites and Electrical Properties

This subsection uncovers profound relationships among terms related to nanocomposites and their electrical properties. Notable associations include “nanocomposite”, “microstructure”, “electrical property”, and “effect”, highlighting the intricate interplay between microstructure and electrical behavior. The research emphasizes a shift toward investigating nanocomposites’ electrical attributes, showcasing this field’s interdisciplinary nature. Crosslinking time emerges as a pivotal factor in shaping nanocomposite electrical characteristics. To delve deeper into these insights, recommended keywords for 2023 research include (i) Nanocomposites, (ii) Electrical Properties, (iii) Microstructure, (iv) Crosslinking Time, (v) Optoelectronic Behaviors, (vi) Interdisciplinary Research, (vii) Fundamental Mechanisms, (viii) Materials Science, (ix) Electrical Engineering, and (x) Analytical Techniques.

#### 4.5.2. Nanocomposites and Mechanical Behavior

This subsection delves into the ongoing developments in nanocomposite research, highlighting the central role of the term “nanocomposite”. It emphasizes the interplay between “microstructure” and “nanocomposite”, offering insights into how structural nuances affect nanocomposite properties. Research extends to understanding the consequences of nanoparticle incorporation, providing comprehensive insights into nanocomposite attributes. Researchers employ advanced analytical tools such as “x-ray diffraction” and “scanning electron microscopy” for deeper insights. Promising areas include optimizing crosslinking times, exploring multifunctional nanocomposites, integrating nanocomposites into specialized sectors like optoelectronics and biocompatible applications, and refining understanding through advanced analytical modalities. To explore these insights further in 2023, use keywords like (i) Nanocomposites, (ii) Mechanical Behavior, (iii) Microstructure, (iv) Nanoparticles, (v) Crosslinking Time, (vi) Multifunctional Nanocomposites, (vii) Advanced Analytical Techniques, (viii) Energy Storage, (ix) Optoelectronics, (x) Biocompatible Nanocomposites, (xi) Material Behaviors, (xii) Morphological Aspects, (xiii) X-ray Diffraction, and (xiv) Scanning Electron Microscopy.

#### 4.5.3. Nanocomposites and Microstructure

This subsection provides a deep understanding of advancements in nanocomposites, focusing on “microstructure” as a pivotal term. It reveals a robust connection between “microstructure” and “nanocomposite”, emphasizing their interdependence at the nanoscale. Microstructure research spans various aspects, including material enhancement, property evaluation, and analytical techniques. The integration of “nanocomposite” into this research landscape underscores an integrative approach to revolutionizing nanocomposite attributes at the nano level. Emerging themes include research into “nanocomposite fiber” and “optical window”, exploring “energy storage properties”, and employing spectroscopic tools for nanocomposite analysis. Promising areas for future exploration include “crosslinking time”, optical properties in optoelectronics and solar cells, nanocomposites in energy storage, and niche terminologies and methodologies. To explore these insights in 2023, use keywords like (i) Nanocomposites, (ii) Microstructure, (iii) Crosslinking Time, (iv) Optical Properties, (v) Energy Storage, (vi) Spectroscopic Techniques, (vii) Nanocomposite Fiber, (viii) Nanocomposite Material, (ix) Microstructural Features, (x) Molybdenum, (xi) PBAT Nanocomposite, (xii) Al_2_O_3_ Tin Nanocomposite, (xiii) Zinc Oxide–Nickel Oxide, (xiv) Carbon Nanofiber Polystyrene Nanocomposite, and (xv) UV–Vis Analytical Spectroscopy.

**Figure 26:**
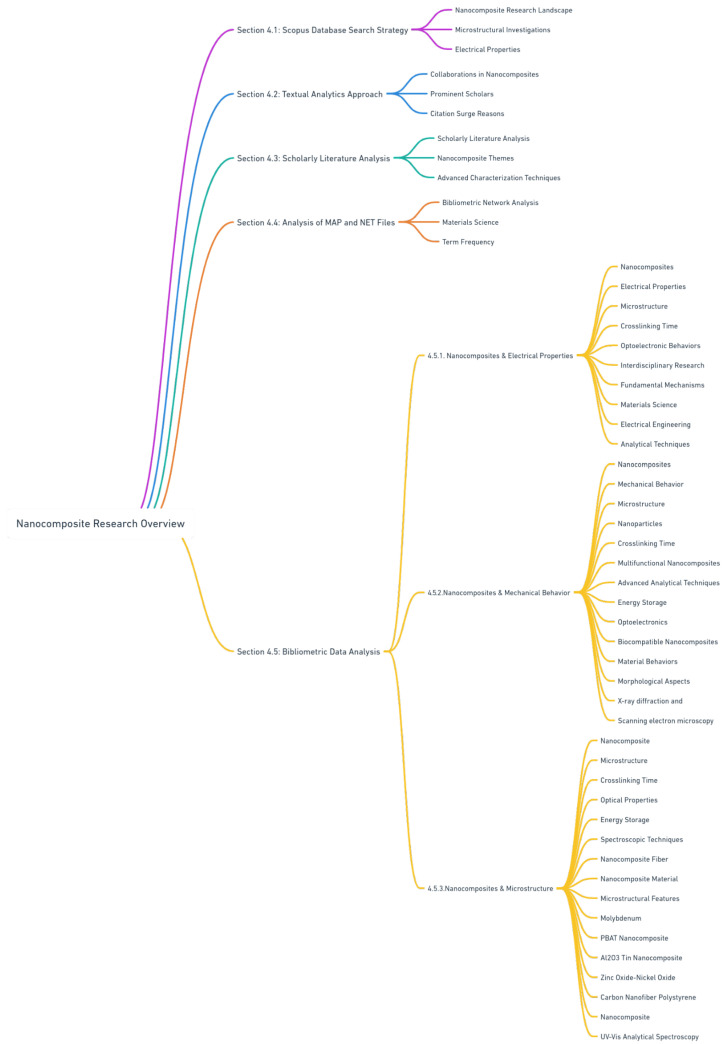
Overview of nanocomposites research.

Section 4 delivers an exhaustive analysis from Section 4.1 to Section 4.5, presenting an expansive examination of nanocomposite studies, as represented in Figure 26.

A review of the Scopus database indicates an extensive compendium comprising 209,012 documents on nanocomposites as of 23 October 2023. This work primarily investigates microstructural elements and their subsequent influence on material behavior. There exists a pronounced interest in deciphering both the electrical and mechanical properties manifested by nanocomposites. The research timeline shows an uptick in the late 1970s, reaching a peak from 2018 to 2022. A growing inclination toward the term “effect” signals an amplified focus on understanding the underlying implications of nanocomposites.

In textual analytics, collaborations prove instrumental in fostering innovation. High citation counts in articles offer pivotal guidance, directing this research field. Emerging trajectories include electromagnetic interference (EMI) shielding, sustainable materials, and significant financial support for nanocomposite research from China. A discernible rise in Chinese language publications in academic discourse becomes evident. Sentiment analysis projects an optimistic direction for nanocomposite research.

Examining scholarly literature highlights many nanocomposite-related topics, encompassing analytical processes, functional characteristics, synthesis approaches, and performance enhancement strategies. Areas of pronounced attention include advanced characterization methods, mechanical properties, biomedical applications, and optoelectronic functionalities.

Delving into the analysis of MAP and NET files through tools such as VOSviewer reveals nuanced relationships between term frequency and Total Link Strength, hinting at potential nascent topics. Patterns become apparent, especially within materials science.

The findings from the Bibliometric Data Analysis and Visualization Report elucidate the nuanced relationship between electrical attributes, mechanical behavior, and microstructural details intrinsic to nanocomposites. The centrality of microstructure and the significance of crosslinking duration in shaping the electrical attributes of these materials are highlighted.

Across Section 4.5.1, Section 4.5.2 and Section 4.5.3, nanocomposites remain the focal point, underscoring their diverse applications and adaptability. The consistent reference to microstructure underscores its paramount role in determining nanocomposite performance. The influence of crosslinking duration recurs throughout, indicating its integral role in modulating material properties. Although there is a unified approach to utilizing advanced analytical methods, specific techniques vary between subsections. For instance, while Section 4.5.1 centers on the electrical attributes of nanocomposites and fields such as optoelectronics and electrical engineering, Section 4.5.2 ventures into the mechanical behavior of these materials, showcasing techniques like X-ray diffraction and scanning electron microscopy.

Conversely, Section 4.5.3 emphasizes UV–Vis Analytical Spectroscopy and presents specific nanocomposite materials like PBAT, Al_2_O_3_ tin, and carbon nanofiber polystyrene, indicating the diverse lenses through which nanocomposites are viewed and analyzed. The range of materials explored, including molybdenum, PBAT, aluminum oxide, tin, zinc oxide, and nickel oxide, signifies the vast scope of nanocomposite research. Data mining and ML methodologies enhance the capability of researchers to identify trends and foresee material behavior, thereby advancing the field of nanomaterials.

To conclude, Section 4 consolidates a plethora of insights, providing a holistic view of the nanocomposite research landscape and setting the stage for future academic endeavors in this versatile domain. Moreover, Table 1, provided at the end of this section, concisely encapsulates these findings alongside a curated list of recommended keywords to guide future research in the dynamic field of nanocomposites.

Table 1 serves as a vital synthesis tool, offering a clear summary of key findings and trends and acting as a roadmap for future research endeavors in nanocomposite research. It delineates critical areas of study and suggests targeted keywords, facilitating a deeper understanding and efficient exploration of the evolving landscape of nanocomposite research. This organized presentation of data highlights the study’s contribution to the scientific community, serving as a valuable reference for researchers aiming to build on these findings or explore new investigative paths in the multifaceted world of nanocomposites.

## 5. Literature Revision Guided by Artificial Intelligence

The insights from Section 4, combined with current trends in nanocomposite research, have led to a revised approach for Scopus database searches. A specific set of keywords, reflective of crucial subtopic connections, was selected to target cutting-edge nanocomposite research in 2023. This new search method involves scanning titles, abstracts, and keywords for the first two themes and using a specified keyword for the third theme, encompassing all document fields. The focus was on publications from 2023, prioritizing articles by citation frequency. The most cited article underwent detailed analysis. Different research goals require modified search strategies.

### 5.1. Nanocomposites and Electrical Properties

This section delves into the intricate relationship between nanocomposites and their electrical properties. It focuses on how these advanced materials interact fundamentally, influencing their electrical characteristics. The examination covers various nanocomposite systems, emphasizing their composition, structure, and functionality. This exploration is crucial for advancing our understanding of how nanocomposites can be optimized for specific electrical applications, particularly in fields requiring enhanced electrical performance. The discussion integrates findings from recent studies, providing a comprehensive view of this area’s current state of knowledge.

#### 5.1.1. Crosslinking Degree and Its Influence on XLPE/OMMT Nanocomposites

Yunzi et al. (2023) [465] studied the complex relationship between crosslinking degree and the mechanical and electrical properties of polyethylene/organic modified montmorillonite (XLPE/OMMT) nanocomposites. This research aligns with current discussions in nanocomposites, electrical properties, and crosslinking duration, revealing intricate interactions in these areas.

The study’s primary goal is to examine how crosslinking degree affects the tensile and dielectric properties of XLPE/OMMT nanocomposites. The team employed various experimental methods to explore this relationship comprehensively.

The methodology includes X-ray diffraction (XRD) for analyzing montmorillonite dispersion and structural changes due to crosslinking. Scanning electron microscopy (SEM) was crucial for observing changes in crystalline morphology and crosslinked networks’ effects on crystal growth. The gel content test provided insights into the evolution of crosslinking degrees over time. Conductance-temperature characteristics, dielectric constant, and dielectric loss tangent tests offered a detailed view of dielectric properties, complemented by power-frequency breakdown field strength analysis using the two-parameter Weibull distribution.

The findings highlight the dynamic nature of XLPE/OMMT nanocomposites under different crosslinking degrees. XRD showed a transition of montmorillonite to an exfoliated state, with reduced crystallinity as crosslinking time increased. The gel content test revealed a complex pattern of crosslinking degree development, peaking at 1520 min. SEM showed that crosslinked networks inhibited crystal growth, leading to a more consistent crystal size distribution in positively crosslinked states. The interaction between OMMT and crosslinked bonds enhanced tensile strength, elastic modulus, and toughness, demonstrating a well-balanced nanocomposite structure.

In summary, this study significantly advances our understanding of the complex relationship between crosslinking degree and the mechanical and electrical properties of XLPE/OMMT nanocomposites. It reveals the multifaceted dynamics of this relationship and highlights the critical role of crosslinking in defining these materials’ macroscopic characteristics. Dong Yunzi and colleagues’ research marks a significant milestone in nanocomposite research, illuminating the complex interplay between nanomaterials, polymer science, and the temporal dynamics of crosslinking processes.

#### 5.1.2. BaTiO_3_ Nanofillers in Polymer Blend Nanocomposites: A Study on PVDF/PMMA/BaTiO_3_

The research on polymer blend nanocomposites (PBNCs) marks a pivotal advancement in developing customizable materials for various applications. Sengwa et al. (2023) [466] conducted a comprehensive study on PVDF/PMMA/BaTiO_3_ nanocomposites. This study is crucial for understanding their applications in modern microelectronic and optoelectronic technologies.

This research examines how BaTiO_3_ nanofillers in varying concentrations affect the properties of the PBNC films. This is especially relevant for creating materials with adjustable properties for diverse technological uses.

The study uses a solution-cast method to prepare PBNC films. These films undergo detailed analysis through several techniques. Scanning electron microscopy (SEM) reveals the uniformity of PBNC films and the morphological changes in PVDF due to increased BaTiO_3_ concentration. X-ray diffraction (XRD) detects electro-active polar β- and γ-phases in PVDF crystallites. Fourier transform infrared (FTIR) spectroscopy corroborates these findings. Differential scanning calorimetry (DSC) explores the thermal properties and melting points of the PBNC materials. Extensive dielectric spectra are thoroughly examined, including complex dielectric permittivity, dielectric loss tangent, and electric modulus spectra. The study also methodically analyzes the electrical conductivity of PBNC films across different frequencies.

The findings of this research demonstrate the significant influence of BaTiO_3_ nanoparticles on PBNC films. Increased BaTiO_3_ concentration leads to high homogeneity and noticeable changes in PVDF’s spherulite morphology. XRD confirms the presence of electro-active polar β- and γ-phases in PVDF crystallites. The optical properties, including UV–Vis absorbance and direct energy band gap, show notable variations related to nanomaterial concentration. The dielectric properties reveal a decrease in the real part of complex dielectric permittivity at higher frequencies and a pronounced chain segmental relaxation process. The electrical conductivity of PBNC films increases with frequency, highlighting their potential in capacitive energy storage and microelectronic devices.

In summary, incorporating BaTiO_3_ nanoparticles into the PVDF/PMMA blend matrix significantly affects the PBNC films’ morphological, structural, thermal, optical, and electrical properties. These materials are suitable for various applications, such as frequency-tunable nanodielectrics, electromagnetic interference shielding, flexible dielectric substrates, thermal insulators, and bandgap-regulated materials in microelectronics, capacitive energy storage, and optoelectronics.

#### 5.1.3. Enhancing Fatigue Life in Aluminum–Graphene Nanocomposites for Power Transmission

Azizi et al.’s 2023 [467] study marks significant progress in comprehending high-cycle fatigue (HCF) in aluminum–graphene nanocomposites, particularly for high-capacity power transmission conductors. Their paper, “Fatigue life prediction of aluminum-graphene nanocomposites: Application to high-capacity conductors”, delves into the Al-0.5 wt% graphene nanoparticle (GNP) composite’s mechanical and electrical attributes, shedding light on its fatigue resilience under various stress levels and temperatures.

The study’s primary goal is to evaluate the fatigue life of the Al-0.5 wt% GNP composite, a promising material for high-capacity conductors. It tackles the challenges of Aeolian vibrations and fatigue failure in commercial aluminum conductors, suggesting graphene nanoparticle integration to boost mechanical and electrical qualities. This research also explores the composite’s fatigue behavior at high temperatures, addressing a vital gap in the current literature.

The authors applied various experimental approaches to the composite at ambient and elevated temperatures, including quasi-static and high-cycle fatigue tests. These tests helped derive material parameters for Basquin’s equation, offering a quantitative perspective of the fatigue behavior. Fractographic analysis using field emission scanning electron microscopy (FESEM) provided insights into failure mechanisms. Raman analysis evaluated the graphene’s characteristics in the composite, and tensile strength tests assessed its mechanical properties.

The findings show a significant enhancement in the fatigue life of the Al-0.5 wt% GNP composite over pure aluminum. At room temperature, the composite shows a remarkable 234% increase in fatigue life at low stress levels and a 44% increase at high stress levels. Elevated temperature tests indicate a 146% improvement at low stress levels and a 130% increase at high stress levels compared to pure aluminum. The graphene nanoparticles are identified as pivotal in delaying crack initiation and slowing crack growth, thus boosting the matrix’s fatigue life.

Microstructural and mechanical tests further validate the composite’s advantages, revealing graphene’s uniform distribution within aluminum and a grain size reduction compared to pure aluminum. This implies that GNPs bolster mechanical strength and induce favorable microstructural modifications in the composite.

Azizi et al.’s research substantially advances nanocomposite materials for high-capacity conductors. The Al-0.5 wt% GNP composite shows superior fatigue life and mechanical properties compared to pure aluminum, crediting graphene nanoparticles for these improvements. This work offers potential for material optimization in power transmission, paving the way for enhanced efficiency and reliability.

#### 5.1.4. Water-Tree Aging in XLPE/OMMT Nanocomposites: The Role of Crosslinking Degree

Nanocomposites, particularly electrical insulation materials, represent a critical research field in evolving modern power systems. Dong et al.’s 2023b article [468], “Effect of Crosslinking Degree on Water-Tree Aging Characteristics of XLPE/OMMT Nanocomposites”, investigates the relationship between the crosslinking degree in crosslinked polyethylene/montmorillonite (XLPE/OMMT) nanocomposites and their electrical properties, focusing on water-tree aging.

Given the increasing voltage levels and challenging conditions in power systems, there is a growing demand for insulation materials with enhanced electrical, thermal, and mechanical properties. Polymer-based nanocomposites, known for their unique structures and exceptional properties, are emerging as effective dielectrics. This article contributes to this field by examining how the crosslinking degree affects water-tree aging in XLPE/OMMT nanocomposites.

The study’s primary aim is to understand how the crosslinking degree influences the water-tree aging characteristics of these nanocomposites. The research team prepared two nanocomposites with different crosslinking degrees and subjected them to accelerated water-tree aging tests. They examined water-tree morphology, measured length, and initiation probability, tested gel content for crosslinking assessment, and analyzed chemical composition changes pre- and post-aging using Fourier transform infrared spectroscopy (FTIR).

The findings reveal a notable effect of crosslinking degree on water-tree aging characteristics. Higher crosslinking degrees result in less dense water-tree morphologies, indicated by smaller fractal dimensions and duty cycles. The study also highlights electrochemical degradation during aging in XLPE/OMMT nanocomposites, offering insights into their complex interactions.

The research employs a diverse range of methods. Accelerated water-tree aging experiments are vital to evaluating performance under simulated conditions. Microscopic analyses, including polarized light and scanning electron microscopy (SEM), are used to observe water-tree morphology and changes in crystal structure. Gel content tests and FTIR analyses aid in assessing crosslinking degree and chemical composition changes during aging.

In summary, Dong et al.’s study significantly enhances our understanding of the relationship between crosslinking degree and water-tree aging in XLPE/OMMT nanocomposites. The research highlights the critical role of optimizing crosslinking degree to achieve desired electrical properties and improve the durability of nanocomposite materials in power systems.

#### 5.1.5. Sn Doping Effects in CdO Nanocomposites: A Laser Ablation Study

Fadhali’s 2023 study, titled “Structural, optical, and electrical characterization of laser ablated CdO_1−x_Sn_x_ nanocomposites” [469], presents an in-depth analysis of CdO:Sn nanocomposites synthesized using laser ablation. Thanks to their distinctive electrical and optical properties, these nanocomposites are notable for their potential in nanophotonics and photovoltaics.

The study’s primary aim is to examine the effects of Sn doping on CdO nanocomposites’ structural, optical, and electrical features. Fadhali achieves this by synthesizing CdO and CdO:Sn nanocomposites with varied Sn doping ratios (5%, 10%, 15%) using laser ablation and conducting thorough characterizations.

This paper’s contributions are diverse. It enhances the understanding of CdO:Sn nanocomposites by exploring their structural, optical, and electrical attributes. The research highlights how Sn doping influences crystallinity in CdO samples. It also investigates the nanocomposites’ size and roughness, showing how these are affected by Sn doping. The study extends to optical characteristics, revealing absorption, transmission, and reflection spectra changes due to Sn doping. It notably examines Sn doping’s impact on the nanocomposites’ energy bandgap, electron concentration, carrier mobility, and resistivity. The paper also explores dielectric properties, noting significant shifts with different Sn doping ratios.

Methodologically, Fadhali employs laser ablation for synthesizing CdO and CdO:Sn nanocomposites. The thin films are deposited using a Q-Switched Nd:YAG laser in a vacuum chamber. Characterization techniques include X-ray diffraction (XRD) for structural analysis, atomic force microscopy (AFM) for surface morphology, UV–Vis spectrophotometry for optical properties, Fourier transform infrared spectroscopy (FTIR) for chemical characterization, and Hall effect measurements for electrical properties.

The results demonstrate various aspects of CdO:Sn nanocomposites. XRD confirms crystalline phases, with cubic structures in CdO and tendencies toward orthorhombic structures in CdO:Sn. AFM shows how Sn doping affects roughness and diameter. Optical measurements reveal plasmonic effects and Sn doping’s influence on spectra. Electrical characterizations indicate improved electron concentration, carrier mobility, and resistivity with increased Sn doping. Dielectric properties show notable variations, indicating peak shifts to higher wavelengths.

In conclusion, Fadhali’s work thoroughly investigates the structural, optical, and electrical properties of CdO:Sn nanocomposites. The findings enrich the understanding of these nanocomposites for potential applications, suggesting that a 10% Sn doping ratio optimally enhances CdO:Sn nanocomposites’ properties.

#### 5.1.6. ZnO/TiO_2_ Nanoparticles in PEO/CMC Nanocomposites: Implications for Flexible Optoelectronics

Ragab’s 2023 study, “Optical, thermal and electrical characterization of PEO/CMC incorporated with ZnO/TiO_2_ NPs for advanced flexible optoelectronic technologies” [470], provides an in-depth analysis of PEO/CMC nanocomposites integrated with zinc oxide (ZnO) and titanium dioxide (TiO_2_) nanoparticles. This research is pivotal in understanding nanocomposite materials, especially for their potential use in flexible capacitors and energy storage systems within flexible optoelectronic technologies.

The main goal of this study is to examine the optical, thermal, and electrical properties of PEO/CMC nanocomposites with various concentrations of ZnO/TiO_2_ nanoparticles. These nanocomposites are synthesized through solution casting, showing promise for advanced flexible optoelectronic applications.

Ragab’s work contributes significantly to the field. The study develops nanocomposite samples of PEO/CMC filled with ZnO/TiO_2_ nanoparticles, broadening the material options for energy devices. It provides essential insights into how ZnO/TiO_2_ nanoparticle concentrations affect the crystallinity and the allowed direct energy gap of the nanocomposite films. The research uses diverse characterization techniques, including FTIR, XRD, UV–Vis analysis, TGA, and impedance analysis, to fully understand the nanocomposite’s properties.

The methodology involves the solution casting method for synthesizing ZnO/TiO_2_ NPs nanocomposites within a PEO/CMC matrix. Characterization techniques include XRD for crystallinity analysis, FTIR for examining metal oxide NPs and PEO/CMC composite interactions, UV/Vis spectroscopy for optical properties, AC conductivity measurements for electrical conductivity, TGA for thermogravimetric analysis, and impedance analysis for dielectric properties investigation.

The study’s findings are notable. Increasing concentrations of ZnO/TiO_2_ NPs reduce the crystallinity of the nanocomposite films. A significant decline in the polymer matrix’s allowed direct energy gap is observed at 7 wt% ZnO/TiO_2_ NP concentration. AC conductivity decreases as ZnO/TiO_2_ NP concentration increases, with correlated barrier hopping (CBH) and non-Debye relaxation processes identified as the dominant conduction mechanisms. The refractive index shows a nonlinear increase with higher ZnO/TiO_2_ NP concentrations, suggesting changes in packing densities and interatomic spacing.

In summary, integrating ZnO/TiO_2_ nanoparticles into the PEO/CMC polymer blend markedly affects its optical, thermal, and electrical characteristics. Changes in crystallinity, energy gap, electrical conductivity, and refractive index are observed, highlighting the potential of PEO/CMC nanocomposites with ZnO/TiO_2_ NPs in flexible optoelectronic devices and energy systems.

#### 5.1.7. Partial Conclusions

The research presented in these six papers, summarized in Table 2, has significantly advanced our understanding of the properties and applications of nanocomposite materials in various technological fields. These studies encompass a range of nanocomposites, including XLPE/OMMT and PVDF/PMMA/BaTiO_3_, and focus on enhancing electrical properties, improving fatigue life, and refining materials for specific uses in power transmission and optoelectronic technologies. They highlight the complex interplay of material composition, structure, and functionality in nanocomposite systems.

These studies utilize various analytical methods, such as X-ray diffraction, impedance analysis, polarized light microscopy, scanning electron microscopy, and Fourier transform infrared spectroscopy. The insights gained from these methods provide a deeper understanding of how nanomaterials interact with polymers and contribute to developing nanocomposite systems. This research demonstrates the potential of nanocomposites to address current and future challenges in materials science and engineering.

Further studies are needed to understand the relationship between crosslinking degree and nanocomposites’ mechanical and electrical properties, especially across various nanomaterial–polymer systems. Research into incorporating graphene nanoparticles into aluminum conductors may yield improvements in mechanical and electrical performance for high-capacity power transmission. Additionally, exploring the fatigue behavior of nanocomposites at high temperatures could provide solutions to fatigue failure issues in commercial aluminum conductors. Future research should also include synthesizing and characterizing CdO:Sn nanocomposites using laser ablation techniques to advance our knowledge of their structural, optical, and electrical properties.

### 5.2. Nanocomposites and Mechanical Behavior

This section methodically examines the intricate relationship between nanocomposites and their mechanical properties. Nanocomposites, composed of a matrix and nanoparticles, demonstrate diverse mechanical behaviors influenced by their microstructure. This segment aims to dissect these behaviors, focusing on how nanoparticle incorporation impacts the overall mechanical performance of these composites. Through a series of studies, it reveals the nuanced effects of various nanoparticles and fabrication techniques on the strength, hardness, and wear resistance of nanocomposites. This exploration advances our understanding of material science and guides the development of nanocomposites for specialized applications, ranging from biomedical implants to structural components.

#### 5.2.1. Enhancement of WE43 Magnesium-Based Nanocomposites through Friction Stir Processing

In nanocomposites, a significant study investigates the mechanical properties and antibacterial behavior of WE43 magnesium-based nanocomposites. Authored by O. Esmaielzadeh et al., the research titled “Investigation of mechanical properties and antibacterial behavior of WE43 magnesium-based nanocomposite” [471] focuses on enhancing WE43 magnesium alloy using ZnO and CuZnO particles. The researchers apply friction stir processing (FSP) to achieve this enhancement.

The study’s primary aim is to explore how FSP affects these nanocomposites’ mechanical and antibacterial properties. Researchers seek to develop biodegradable orthopedic implants from magnesium alloys, which align with human bone regarding mechanical properties. They use FSP to refine grain and evenly distribute secondary phase particles, enhancing the alloy’s strength.

This paper demonstrates the increased mechanical strength of WE43 magnesium alloy post-FSP. It evaluates the microstructure and mechanical properties of the alloy when reinforced with ZnO and CuZnO nanoparticles. Additionally, it assesses the antibacterial properties of these composites against bacteria like *S. aureus* and *E. coli*.

The methodology includes synthesizing ZnO powders, preparing WE43 alloy plates, and executing FSP with tools made of hardened tool steel. Researchers insert reinforcement particles using groove and hole methods. They use field emission scanning electron microscopy (FESEM) for microstructural characterization and test compressive strength and stress–strain relations for mechanical properties. Antibacterial properties are tested using disk samples.

Results show FSP’s effectiveness in reducing grain size and redistributing secondary phase particles in the WE43 alloy. Nano-sized reinforcements distribute more efficiently within the matrix than micro-sized ones. No chemical reaction between reinforcement particles and the alloy is evident. FSP-processed composites exhibit higher microhardness compared to untreated and FSP-processed samples. Furthermore, adding ZnO and CuZnO particles improves the composites’ antibacterial capabilities, especially against *E. coli*.

In conclusion, the research highlights FSP’s role in enhancing the mechanical and antibacterial properties of WE43 magnesium-based nanocomposites. This systematic study contributes to our understanding of these materials and advances the development of materials with superior mechanical strength and antibacterial functionality.

#### 5.2.2. Role of Crosslinking in XLPE/OMMT Nanocomposites

The study of nanocomposites, mainly focusing on their mechanical behavior and dimensional mesh structures, has significantly increased scientific interest. Researchers Gao Dongyunzi et al. [465] have contributed notably to this field with their research on crosslinked polyethylene/organic montmorillonite (XLPE/OMMT) nanocomposites. Their paper examines the complex relationship between the degree of crosslinking and these materials’ tensile and dielectric properties.

This research aims to deeply understand how varying degrees of crosslinking affect the mechanical and electrical properties of XLPE/OMMT nanocomposites. Such understanding is critical for optimizing the crosslinking process to enhance material performance.

The authors apply advanced analytical techniques to study the nanocomposites’ microstructure. They use X-ray diffraction (XRD) to examine montmorillonite dispersion and structural changes in the samples. Scanning electron microscopy (SEM) investigates changes in crystalline morphology. Gel content tests measure the degree of crosslinking, while the study of tensile properties focuses on plastic and elastic deformation. Dielectric properties are evaluated through conductance-temperature characteristics, dielectric constant, and dielectric loss tangent tests. The researchers also applied the two-parameter Weibull distribution to analyze power-frequency breakdown field strength in depth.

The study reveals that the degree of crosslinking significantly affects crystal size uniformity and crystallinity in XLPE/OMMT nanocomposites. Optimal crosslinking, identified within a 15–20 min window, enhances tensile strength, elasticity, and toughness. The interaction between crosslinking and OMMT also improves electrical conductivity and dielectric properties. However, due to crystal structure disruption, excessive crosslinking beyond 20 min adversely affects tensile performance.

The paper’s findings elucidate the complex interplay between crosslinking degree, OMMT dispersion, and electrical conductivity in nanocomposites. This research deepens our fundamental understanding of these interactions and offers practical insights for achieving optimal crosslinking for better mechanical and electrical properties in XLPE/OMMT nanocomposites.

In conclusion, the research highlights the critical role of crosslinking degree in determining the mechanical and electrical capabilities of XLPE/OMMT nanocomposites. Identifying a beneficial crosslinking period of 15–20 min provides a valuable guideline for future work in nanocomposite property optimization. The study also emphasizes the importance of the interplay between OMMT and the polymer matrix in enhancing these advanced materials’ electrical and mechanical characteristics.

#### 5.2.3. Al_2_O_3_ Reinforcement in Brass Matrix Nanocomposites

The study by Shayan Memar et al. [472] advances the understanding of nanocomposites, specifically Al_2_O_3_/brass matrix nanocomposites. Their research, “An evaluation on microstructure, wear, and compression behavior of Al_2_O_3_/brass matrix nanocomposites fabricated by stir casting method”, focuses on using stir casting to create leaded brass composites reinforced with Al_2_O_3_ nanoparticles.

This research aims to evaluate the impact of Al_2_O_3_ nanoparticle reinforcement on the mechanical properties of these nanocomposites. While copper matrix composites are well studied, brass matrix composites, particularly those reinforced with Al_2_O_3_, have yet to be explored more. This study addresses this gap, examining mechanical properties and wear behavior through stir casting.

The paper’s contributions are multifaceted. It explores how Al_2_O_3_ nanoparticles affect the nanocomposites’ mechanical properties, focusing on changes in microhardness and toughness. The study also investigates wear and compression behavior, noting a shift in wear mechanism from cutting to plow abrasive wear with nanoparticle addition. FESEM and XRD analyses confirm the nanoparticles’ uniform distribution within the brass matrix. This research enhances the understanding of wear-resistant materials.

The methodology encompasses material preparation with leaded brass ingot and Al_2_O_3_ nanoparticles, using stir casting for fabrication. Pre-treatment includes ball milling Al_2_O_3_ nanoparticles with copper microparticles. The microstructural analysis uses field emission scanning electron microscopy (FESEM), while mechanical testing includes Vickers hardness and microhardness measurements, along with compressive and wear testing following ASTM G99 standards. Porosity and chemical composition analyses are also crucial.

Results from this study show that FESEM images confirm uniform Al_2_O_3_ nanoparticle distribution in the brass matrix. The nanocomposites demonstrate improved microhardness and toughness compared to unreinforced brass. However, nanocomposites exhibit a higher wear rate, attributed to nanoparticle addition. The wear mechanism shifts from cutting to plow abrasive wear. The composites’ porosity remains below 5%, with density increasing and porosity decreasing due to nanoparticle addition. Wear testing indicates slightly higher specific wear rates and friction coefficients (COF) for nanocomposites than leaded brass.

In conclusion, Al_2_O_3_ nanoparticles significantly enhance the microhardness, toughness, and elongation percent of leaded brass nanocomposites. The nanoparticles obstruct dislocation movement, increasing microhardness. Toughness and elongation improvements relate to a reduction in the β-phase percentage. While wear rates and COF are marginally higher in nanocomposites, the study highlights the overall positive effect of Al_2_O_3_ reinforcement on the mechanical properties of brass matrix nanocomposites.

#### 5.2.4. Zinc Oxide Nanoparticles in PLA/PCL Bionanocomposites

Amir Babaei et al.’s work on polylactic acid/polycaprolactone (PLA/PCL) bionanocomposites stands out in nanocomposite research [473]. Their study, “Polylactic acid/polycaprolactone bionanocomposites containing zinc oxide nanoparticles: Structure, characterization and cytotoxicity assay”, thoroughly examines the structural, thermal, mechanical, and biocompatible characteristics of these bionanocomposites.

The study aims to explore biodegradable polymers, particularly PLA, as alternatives to petroleum polymers, addressing environmental concerns. It investigates the integration of PCL into PLA to improve brittleness and thermal stability. Additionally, it pioneered zinc oxide nanoparticles (ZnO-NPs) in PLA/PCL blends, recognizing their potential to enhance antimicrobial, photocatalytic, and biocompatible properties.

The paper’s contributions are diverse. The team synthesizes ZnO-NPs using a hydrothermal method. Analyses by FESEM, XRD, and FTIR confirm these nanoparticles’ hexagonal structure and size. When added to the PLA/PCL blend, ZnO-NPs induce various structural improvements. The study examines morphological changes (via FESEM), increased crystallinity and melting temperature (via DSC analysis), enhanced tensile strength and modulus, altered rheological behavior indicating a 3D network structure, and accelerated degradation under UV light. It also evaluates the cytotoxicity of the nanocomposites on fibroblast cells, crucial for biomedical uses.

Methodologically, the study employs FESEM, XRD, and FTIR for synthesis and characterization, confirming ZnO-NPs’ structure and dispersion in the PLA/PCL blend. DSC and tensile testing analyze thermal and mechanical aspects, while rheological properties are examined using a rheometrics mechanical spectrometer. The cytotoxicity assay uses MTT and acridine orange fluorescence staining to evaluate the bionanocomposites’ impact on fibroblast cells.

Results show successful ZnO-NP synthesis and uniform PLA/PCL blend dispersion. ZnO-NPs increase PLA’s crystallinity and melting temperature and improve tensile strength and modulus. Rheological analyses indicate the development of a 3D-network structure. The bionanocomposites also show accelerated photochemical degradation, suitable for light-responsive applications. Notably, the cytotoxicity assay shows no harmful effects on fibroblast cells, suggesting biomedical application potential.

In conclusion, this research is pioneering in incorporating ZnO-NPs into PLA/PCL blends, highlighting its structural, thermal, mechanical, and biocompatible properties. This study deepens our understanding of these bionanocomposites and opens avenues for their use in eco-friendly and biomedical fields.

#### 5.2.5. Aluminum Oxyhydroxide in Dental Nanocomposites

Nanocomposite materials are crucial in dental advancements, as shown in Savita Kumari et al.’s study [474], “Enhanced physical and mechanical properties of resin added with aluminum oxyhydroxide for dental applications”. This research focuses on adding aluminum oxyhydroxide (AlOOH) to resin composites to improve their mechanical and tribological properties for dental restorations.

The study aims to overcome the mechanical limitations of polymethyl methacrylate (PMMA), widely used in denture bases. Despite PMMA’s transparency and heat resistance, its mechanical strength is insufficient for high-stress dental applications. The research explores resin-based composites (RBCs) with AlOOH, a non-toxic, heat-resistant metal oxide filler that strengthens polymers at room temperature, enhancing PMMA’s mechanical performance while maintaining biocompatibility.

This paper makes significant contributions. The team develops resin composites with AlOOH, enhancing mechanical and tribological properties for dental use. They use a heat-curing method to synthesize these composites, showing the process’s scalability for incorporating AlOOH into the PMMA-ZrO_2_ matrix. The study uses XRD, FTIR, SEM, EDAX, and Nanozetasizer to characterize the nanocomposites’ structural and surface properties comprehensively. Optical and mechanical testing, including UV–Vis spectra analysis and Universal Testing Machine assessments, provide insights into the composites’ optical behavior and mechanical properties.

The study finds that adding AlOOH to the PMMA-ZrO_2_ matrix increases crystallinity and crystallite size, affecting optical behavior as seen in direct and indirect band gap determination. The PZA15 composite, in particular, shows improved mechanical performance, with enhanced compressive and flexural strength and a reduced friction coefficient. Biocompatibility assessments using MTT assay confirm the PZA15 composite’s suitability for dental applications. Although water absorption slightly increases, it does not significantly affect performance.

In conclusion, this research enhances the understanding of nanocomposite materials in dental applications, especially denture fabrication. AlOOH’s addition effectively enhances PMMA’s mechanical strength and tribological properties, paving the way for more robust, more biocompatible denture materials.

#### 5.2.6. Partial Conclusions

The array of studies presented in Table 3 offers substantial progress in understanding the mechanical behaviors of nanocomposites. These investigations highlight the intricate relationship between nanoparticle types, fabrication methods, and the resulting mechanical characteristics of nanocomposites.

Key insights include the essential role of friction stir processing (FSP) in enhancing the mechanical and antibacterial properties of WE43 magnesium-based nanocomposites. This method is crucial for producing materials that exhibit superior strength and antibacterial capabilities. In the realm of XLPE/OMMT nanocomposites, the degree of crosslinking is identified as a critical factor influencing their mechanical and electrical properties, with an optimal crosslinking period of 15–20 min noted for optimal enhancement.

Incorporating Al_2_O_3_ nanoparticles into brass matrix nanocomposites significantly improves their microhardness, toughness, and elongation. However, this enhancement may lead to modest increases in wear rates and friction coefficients. Despite these challenges, the overall benefits of Al_2_O_3_ reinforcement are clear.

Additionally, integrating ZnO nanoparticles into PLA/PCL blends yields promising results regarding structural, thermal, mechanical, and biocompatibility attributes. This development opens new pathways for applying these composites in environmentally friendly and biomedical contexts. In dentistry, adding aluminum oxyhydroxide (AlOOH) to PMMA enhances mechanical strength and tribological properties, marking a step toward more robust and biocompatible dental materials.

Looking forward, future research should address various aspects of nanocomposites. Investigating the long-term stability and corrosion resistance of WE43 magnesium-based nanocomposites post-FSP is vital, particularly for their potential use in biodegradable orthopedic implants. Optimizing the crosslinking degree in XLPE/OMMT nanocomposites is essential to balance mechanical and electrical properties. Understanding the impact of water absorption on the performance of these materials, especially in dental applications, remains crucial. Additionally, research should focus on understanding and improving wear mechanisms in nanocomposites, particularly those reinforced with Al_2_O_3_ nanoparticles. Efforts to reduce wear rates and friction coefficients while maintaining enhanced microhardness and toughness will be pivotal in advancing the field of nanocomposites.

### 5.3. Nanocomposites and Microstructure

This section delves into the pivotal role of microstructure in nanocomposites, focusing on how crosslinking time impacts their surface characteristics. The scope of this discussion encompasses various functionalities, notably energy storage, within specified material systems, exemplified by the presence of “manganese tungstate” with enhanced performance criteria. Additionally, the terms “zoi” (zone of inhibition) and “UV-Vis analytical spectroscopy” signify a keen interest in exploring the antimicrobial properties of nanocomposites through spectroscopic analysis. Furthermore, the term “pure CF” directs our attention to the significance of pure carbon fibers within nanocomposite architecture.

To facilitate efficient navigation of the extensive literature corpus, we have provided a set of keywords that serve as a strategic framework. These keywords are intended to guide researchers in identifying seminal studies that comprehensively understand the current state of the art in nanocomposites. Each set of keywords has been meticulously designed to highlight research at the forefront of this field, emphasizing interdisciplinary methodologies, cutting-edge characterization techniques, and focused material investigations.

#### 5.3.1. Modulation of Electro-Optical Properties in PDLC Films Using MWCNT-Loaded Reticular Nanofiber Films

In their recent study, Miao et al. (2023) [475] conducted a comprehensive investigation focused on the modulation of electro-optical properties in polymer-dispersed liquid crystal (PDLC) films. They achieved this modulation by incorporating multiwalled carbon nanotube (MWCNT) loaded reticular nanofiber films. This research is particularly significant within PDLC technology, where electro-optical performance is critical.

The study’s primary objective was to enhance the electro-optical properties of PDLC films by addressing the challenges associated with conventional methods of incorporating MWCNTs. While PDLC films find extensive application in displays and smart windows, their improvement has historically involved the integration of nanomaterials and adjustments to polymerization conditions. However, the incorporation of MWCNTs, while holding promise for enhancing electro-optical properties, has faced issues such as agglomeration and a reduction in device lifespan. To overcome these limitations, the authors propose a novel approach: utilizing MWCNT-loaded reticular nanofiber films to optimize the interaction between MWCNTs and PDLC.

The contributions of this research are multifaceted. Firstly, adding MWCNT-loaded nanofibers significantly enhances the electro-optical properties of PDLC films, resulting in improved stability and a heightened contrast ratio. Moreover, the study explores the influence of different polymer monomer ratios, focusing on the multifunctional monomer HPMA, on the overall performance of PDLC films. The findings demonstrate that an increase in HPMA content reduces the polymer network’s size, ultimately improving electro-optical properties.

The methodological framework employed in this study is both robust and thorough. PDLC films were fabricated using the polymerization-induced phase separation (PIPS) method, incorporating a prepolymer, liquid crystal, and MWCNT-loaded reticular nanofiber films. Subsequently, advanced techniques such as scanning electron microscopy (SEM), polarized light microscopy (POM), transmission electron microscopy (TEM), and electro-optical curve analysis were employed to analyze the microstructure and electro-optical profiles of various PDLC samples.

Furthermore, the electro-optical properties of the PDLC films were meticulously evaluated using a liquid crystal comprehensive parameter meter (LCT-5016). This comprehensive analysis provided insights into threshold voltage, drive voltage, contrast ratio, off-state transmittance, and on-state response time. Additionally, the study delved into the morphological aspects of PDLC films and MWCNTs using SEM, TEM, and POM.

The results underscore the positive impact of MWCNT-loaded reticular nanofiber films on PDLC films. This impact is evident in the lower driving voltages required, higher contrast achieved, and faster response times observed. The proportion of liquid crystals in PDLC films emerged as a critical factor influencing transmittance, threshold and saturation voltages, and contrast. Additionally, adjusting the multifunctional monomer HPMA ratio within the polymer matrix proved instrumental in controlling the size of the polymer network and, consequently, enhancing electro-optical properties.

In conclusion, Miao et al. (2023) present a significant advancement in PDLC films by exploring MWCNT-loaded reticular nanofiber films. This research sheds light on the achieved electro-optical enhancements and provides valuable insights into the influence of polymer monomer ratios on the overall performance of PDLC films. These findings pave the way for developing PDLC-based devices with superior response times, enhanced electro-optical properties, and prolonged stability, expanding their potential applications in optical windows, displays, energy storage, and flexible devices.

#### 5.3.2. Enhancing Nanocomposites with Well-Crystallized Zinc Oxide Nanorods and Chitosan/PVP Polymers

In the study conducted by Alghamdi and Rajeh (2023), [476] a comprehensive investigation was undertaken to analyze various parameters, including structural, optical, thermal, and electrical characteristics, of nanocomposites formed by blending well-crystallized zinc oxide nanorods (ZnO NRs) with chitosan/polyvinyl pyrrolidone (Cs/PVP) polymers. The primary objective of this research was to enhance our understanding of the synergistic effects of combining ZnO nanostructures with Cs/PVP blends and elucidate their potential applications in energy storage devices and thin-film solar cells.

This study aligns with the increasing interest in environmentally friendly and renewable materials for electrochemical devices. It introduces biopolymer-based solid polymer electrolytes (SPEs), derived from natural sources such as chitosan and polyvinylpyrrolidone, as a sustainable alternative. Notably, incorporating ZnO nanostructures into these biopolymer matrices represents a novel approach, considering the favorable properties of ZnO nanoparticles. The investigation aimed to bridge existing knowledge gaps by unraveling the impact of ZnO nanostructures on the structural, optical, thermal, and electrical characteristics of Cs/PVP blends.

The contributions of this research are manifold. Firstly, it delves into the structural aspects, confirming the well-crystallized nature of ZnO NRs through X-ray diffraction (XRD) analysis while noting an increase in amorphous character within the polymer composites. The dimensions of the ZnO nanorods, which play a crucial role in their optical and electrical properties, were determined through transmission electron microscopy (TEM), revealing lengths ranging from 200 to 300 nm and diameters ranging from 40 to 80 nm.

The optical characteristics of the nanocomposites were comprehensively investigated, revealing a narrowing of the optical bandgap and an increase in the Urbach energy, extinction coefficient, refractive index, and optical conductivity upon incorporating ZnO NRs into the polymer matrix. Photoluminescence (PL) spectra further highlighted a distinctive photoemission peak around 470 nm in films made of polymer nanocomposites, emphasizing the intriguing optical properties of the Cs/PVP-ZnO system.

Regarding electrical properties, the Cs/PVP-ZnO nanocomposite exhibited elevated ionic conductivity at normal temperature, indicating its potential for use in energy storage devices. Electric modulus and dielectric permittivity studies provided valuable insights into conductivity relaxation and charge storage characteristics, shedding light on the nuanced electrical behavior of the nanocomposite.

The results portrayed a comprehensive picture of the Cs/PVP-ZnO nanocomposite, emphasizing its improved structural, optical, thermal, and electrical characteristics. Notably, the observed reduction in crystallinity, blue shift in optical reflectance onsets, and enhanced thermal stability further underscored the suitability of this nanocomposite for applications in thin-film solar cells and energy storage devices.

In conclusion, Alghamdi and Rajeh (2023) have significantly advanced our understanding of nanocomposites by systematically investigating the Cs/PVP-ZnO system. Their findings contribute to fundamental knowledge regarding the structural and optical modifications induced by ZnO NRs in the Cs/PVP blend and highlight the enhanced electrical conductivity and dielectric properties. This positions the Cs/PVP-ZnO nanocomposite as a promising candidate for various energy storage applications.

#### 5.3.3. High-Entropy Nanofibers Transforming the Energy Storage Performance of Polymer Composites

Polymer dielectrics, though flexible and scalable, encounter challenges in high-temperature environments, prompting the inclusion of fillers to enhance their properties. This study by Dou et al. (2023) [477] builds upon the high-entropy approach, originally designed to boost the energy density of ceramic film capacitors to create high-entropy nanofibers. The primary goal is to transform the energy storage performance of polymer composites. The central focus is on systematically investigating how these high-entropy nanofibers impact the microstructure and properties of the polymer matrix, particularly emphasizing dielectric breakdown properties and cyclic charge–discharge reliability.

This research offers several noteworthy contributions. Firstly, it introduces a high-entropy approach for synthesizing nanofibers, presenting a unique strategy to control the microstructure of ceramic fillers within polymer composites. This approach significantly improves dielectric breakdown properties and cyclic charge–discharge reliability, directly influencing fatigue resistance and overall composite reliability. The study imparts valuable insights into designing high-performance dielectric polymer nanocomposites by manipulating filler characteristics, such as crystal phase, grain size, and the amorphous-like region.

Regarding methodology, high-entropy nanofibers are synthesized using an electrospinning method followed by calcination, resulting in 1D ceramic nanofibers with diverse crystal structures. Rigorous analysis of dielectric properties and energy storage performance of resulting polymer composites is conducted through various techniques. Morphology characterization utilizes optical and SEM images, while dielectric performance is evaluated through dielectric constant and dissipation factor measurements, considering their frequency and temperature dependencies. Dielectric breakdown properties are assessed using a two-parameter Weibull statistical distribution function, and P-E loops are employed to measure the polarization-electric field behavior of the composites.

The results demonstrate the effectiveness of high-entropy nanofibers in enhancing the dielectric and energy storage performance of polymer composites. Adding high-entropy nanofibers results in a refined microstructure characterized by an increased proportion of the amorphous-like phase and reduced grain size, leading to a smoother surface and improved properties. Notably, the PEIHEnf composite film exhibits superior cycling stability and fatigue resistance compared to pure polymer films, displaying minimal performance variation in energy density and efficiency.

In conclusion, Dou et al. (2023) have pioneered the utilization of high-entropy nanofibers to revolutionize the energy storage performance of polymer nanocomposites. Their findings pave the way for developing advanced energy storage materials, influencing the design and performance of electric devices and power systems. High-entropy nanofibers emerge as promising candidates for the next generation of dielectric polymer nanocomposites.

#### 5.3.4. Surface Decoration of MnNiWO_4_ Nanostructures on Carbon Nanofiber for Photocatalytic Dye Removal

In the study titled ‘Surface decoration of MnNiWO_4_ nanostructures on carbon nanofiber to build nanocomposites towards the removal of anionic azo and cationic dyes under light illumination,’ conducted by Sai Kumar A. et al. (2023) [478], the primary focus is on the synthesis and characterization of MnNiWO_4_ hybrid nanostructures attached to carbon nanofibers (CNFs). The overarching goal is to enable the photocatalytic removal of anionic azo and cationic dyes when exposed to light. This research addresses the increasing concerns regarding water contamination, particularly from industrial activities such as the textile sector, and aims to overcome the limitations of traditional water treatment methods. Leveraging the cost-effectiveness and environmental friendliness of photocatalysis, the researchers explore the potential of MnNiWO_4_/CNF hybrid nanocomposites for efficient dye removal.

This study makes multifaceted contributions. Primarily, it involves the synthesis and detailed characterization of MnNiWO_4_ hybrid nanostructures on CNFs, achieved through hydrothermal and wet impregnation methods. The investigation encompasses an array of factors, including the crystal structure, optical and electrical properties, microstructure, elemental composition, and structural characteristics of the prepared photocatalysts. To achieve this, a range of advanced techniques, such as XRD, UV–Vis DRS, FL, FESEM, EDS, XPS, and TEM, are employed to unravel the factors influencing the superior photocatalytic performance of the MnNiWO_4_/CNF hybrid nanocomposite. These factors include size, surface charge, electronic effects, and effective charge-transfer abilities.

The methodology section provides a comprehensive overview of the synthesis process, involving the creation of manganese tungstate (MnWO_4_) nanosheets, nickel tungstate (NiWO_4_) nanoparticles, and manganese nickel tungstate (MnNiWO_4_) hybrid nanostructures via a hydrothermal method. The attachment of these hybrid nanostructures to CNFs is achieved through a wet impregnation method. Extensive characterization techniques, including XRD, UV–Vis DRS, FL, FESEM, EDS, XPS, and TEM, are employed to scrutinize the crystal structure, optical and electrical properties, microstructure, elemental composition, and structural characteristics of the photocatalysts. Photocatalytic activity tests assess the degradation efficiency of the synthesized samples toward anionic azo dyes (Orange II sodium salt) and cationic dyes (methyl orange and methylene blue) under light illumination, utilizing a commercial solar simulator as the light source.

The results of the study reveal the exceptional performance of the MnNiWO_4_/CNF hybrid nanocomposite, with an 85% degradation efficiency for the anionic azo dye Orange II sodium salt under light illumination, surpassing the performance of other photocatalysts. Additionally, the hybrid nanocomposite exhibits high degradation efficiencies for anionic dye methyl orange (77%) and cationic dye methylene blue (61%). The photocatalytic degradation process follows pseudo-first-order reaction kinetics. Distinctive characteristics of the MnNiWO_4_/CNF hybrid nanocomposite, such as a substantial red shift in absorption, a reduced energy band gap, and higher crystallinity, contribute to its superior performance.

In conclusion, Sai Kumar A. et al. (2023) provide valuable insights into the development of MnNiWO_4_/CNF hybrid nanocomposites as efficient photocatalysts for the removal of anionic azo and cationic dyes. Their contributions extend to the synthesis and comprehensive characterization of these nanocomposites, elucidating the key factors influencing their superior photocatalytic performance. These findings hold significant promise for addressing water contamination challenges stemming from industrial dye discharge, offering a promising avenue for advanced water treatment technologies.

#### 5.3.5. Synthesis and Characterization of ZnO:GO/rGO Composite Thin Films for Energy Harvesting

The investigation carried out by Joshi et al. (2023) [479] focuses on the synthesis and characterization of graphene oxide (GO) and reduced graphene oxide (rGO) and their impact on the structural and optical properties of zinc oxide (ZnO) thin films. This study’s primary objective is to assess these nanocomposites’ potential for energy harvesting applications, particularly in the context of photovoltaic technology and dye-sensitized solar cells (DSSCs).

The paper’s objectives are multifaceted. Firstly, it explores the synthesis and characterization of GO and rGO, followed by an investigation into their influence on ZnO thin films. These thin films are deposited on glass substrates using the practical and straightforward spin coating method. The structural properties of the films are analyzed using Fourier transform infrared spectroscopy (FTIR) and X-ray diffraction (XRD), revealing the presence of oxygen functionalities and a hexagonal wurtzite crystal structure.

The study further examines the optical properties of the films, demonstrating their high transmittance in the UV–visible region, making them suitable for optoelectronic applications. Moreover, the electrical properties are analyzed, revealing that an increase in GO and rGO weight percentage leads to an increase in crystallite size and a decrease in resistivity. Additionally, the surface microstructure of the GO and rGO-inserted samples is scrutinized using high-resolution electron microscopy.

The investigation resulted in several key findings regarding composite thin films’ structural, optical, and electrical properties. Firstly, concerning structural properties, both undoped and doped ZnO thin films exhibited a hexagonal wurtzite crystal structure, as confirmed through X-ray diffraction (XRD) analysis. Notably, the insertion of graphene oxide (GO) and reduced graphene oxide (rGO) led to an increase in crystallite size, consequently enhancing the conductivity of the thin films.

Moving on to optical properties, the study revealed a noteworthy enhancement in the optical transmittance of rGO/ZnO thin films due to the increased presence of sp^2^ carbon domains in rGO. This improved optical property renders these films suitable for utilization as photoelectrodes in solar cells and various optoelectronic devices.

Lastly, the investigation into electrical properties demonstrated a reduction in resistivity upon adding GO and rGO in ZnO. This decrease in resistivity signifies an increase in conductivity. The observed improvement in conductivity can be attributed to the interface formed between GO/rGO and the ZnO surface, which facilitates the flow of charge carriers. Furthermore, this reduction in resistivity correlates with the increase in crystallite size obtained from the XRD results, further underlining the suitability of these composite thin films for applications as photoelectrodes in energy devices.

These findings suggest that ZnO:GO/rGO composite thin films exhibit favorable structural, optical, and electrical properties, positioning them as promising candidates for photoelectrodes in energy harvesting applications.

In conclusion, the study underscores the significant contributions of incorporating GO and rGO into ZnO thin films for energy harvesting applications. The optimized structural and optical properties and improved electrical characteristics make these nanocomposites potential candidates for photoelectrodes in solar cells and other optoelectronic devices, contributing to the advancement of sustainable and renewable energy solutions.

#### 5.3.6. Promoting Cell Growth with Laser-Synthesized Magnesium Nanoparticles for Tissue Engineering

The article by Nyabadza et al. (2023) [480] is centered on producing magnesium nanoparticles (MgNPs) using laser ablation techniques. These MgNPs are subsequently employed for immobilizing amino acids and enzymes to enhance biochemical reactions and enable targeted delivery to tissues.

The primary objective of this study is to investigate the growth-promoting effects of MgNPs on human dermal fibroblast cells, along with assessing the dynamic reciprocity environment created by these MgNPs. The immobilization of amino acids and enzymes onto the MgNPs is achieved through adsorption, resulting in composites characterized by highly connected needle-like structures, as observed through field emission scanning electron microscopy (FESEM).

This paper offers noteworthy contributions, detailing the fabrication of MgNPs through laser ablation and showcasing their potential for tissue engineering applications, evidenced by their cell growth-promoting effects and improved cell adhesion. Furthermore, the study sheds light on the adsorption mechanism for immobilizing amino acids and enzymes onto MgNPs and visualizes the resulting composites through FESEM.

The methods employed in this study encompass the laser ablation technique for MgNP synthesis, nanoparticle characterization utilizing Dynamic Light Scattering (DLS) and FESEM with energy-dispersive X-ray (EDX) analysis, and the examination of optical properties and concentration via UV–Vis and Fourier transform infrared (FTIR) spectroscopy. Additionally, an image processing algorithm developed in MATLAB aids in quantitatively extracting data from FESEM images. The study involves physical and chemical elution processes, analyzing microstructural changes in MgNP-trypsin and MgNP-glutamine composites before and after elution. Specific details about Pulsed Laser Ablation in Liquid (PLAL) are provided, encompassing parameters such as laser fluence, pulse width, repetition rate, and laser scan speed.

The results of this study demonstrate the promising cell growth-promoting effects and improved cell adhesion achieved by the synthesized MgNPs. FESEM visualizations reveal the presence of highly interconnected needle-like structures in MgNP composites, and the microstructure of MgNP-trypsin composites remains stable post-elution. At the same time, morphological changes are observed in MgNP-glutamine composites. Successful bonding of MgNPs with trypsin and glutamine suggests potential applications in enzyme and amino acid delivery.

In conclusion, Nyabadza et al. (2023) establish the potential utility of laser-synthesized MgNPs for immobilizing and delivering enzymes and amino acids, with implications for various biomedical applications. Their research underscores the importance of MgNPs in creating dynamic reciprocity environments, enhancing cell adhesion, and providing a foundation for future investigations, including microbial lysis efficacy and biocompatibility studies.

#### 5.3.7. Enhancing Bio-Based PLA Composites with Graphene-Based Materials and Wheat Straw

The article authored by Chougan et al. (2023) [481] delves into the enhancement of wheat straw’s compatibility with a polylactic acid (PLA) matrix through the utilization of graphene-based materials (GBMs) for surface functionalization. This endeavor aims to elevate PLA bio-based composites’ mechanical and thermal performance. The study compares composites with and without GBM surface functionalization of straw particles, demonstrating superior thermal stability, flexural strength, tensile strength, and tensile toughness in the former, signifying improved interfacial bonding between straw and PLA matrix.

The primary objective of this paper is to investigate the potential of graphene-based materials as surface-modifying agents to enhance the performance of bio-based PLA composites. The authors built upon their prior research, which established wheat straw as a partial replacement for the PLA polymer matrix. This study introduces a physical pre-treatment (HthS) to modify wheat straw’s surface chemical functional groups, reducing hydrophobic components. Notably, the research demonstrates the effectiveness of GBMs in functionalizing the surface of pretreated straw particles, resulting in noteworthy improvements in the mechanical and physical properties of straw-PLA composites.

The employed methods encompass dry-mixing and pre-heating PLA polymer pellets and wheat straw particles, with and without GBM surface functionalization, followed by hot-pressing. Tensile and flexural experiments, morphology assessments, and tests for dimension stability (thickness swelling and water absorption) contribute to the comprehensive evaluation of the modified composites. The integration of the area under the stress–strain curve is employed to assess tensile toughness.

The results confirm the positive impact of GBM surface functionalization on wheat straw, leading to improved mechanical and thermal performance of PLA bio-based composites. Graphene oxide-functionalized straw particles, in particular, exhibit significant enhancements, with a 27% increase in flexural strength, a 66% increase in tensile strength, and a remarkable 322% increase in tensile toughness compared to control samples. Morphological assessments affirm improved straw/PLA matrix interfacial bonding induced by GBMs, while water absorption and thickness swelling tests underscore the improved dimension stability of functionalized straw-PLA composites.

In conclusion, the study demonstrates the efficacy of surface modification of wheat straw with GBMs to enhance bio-based PLA composites’ performance significantly. The findings have implications for developing sustainable and high-performance materials, bridging the gap in the literature regarding the use of graphene-based materials for surface functionalization in wheat straw bio-based composites.

#### 5.3.8. Improving Carbon Foam with Multiwalled Carbon Nanotubes and Functionalized Nanodiamonds

The article authored by Aslam et al. (2023) [482] delves into the enhancement of pitch-derived carbon foam (CF) through the incorporation of multiwalled carbon nanotubes (MWCNTs) and functionalized nanodiamonds (FNDs). The study, rooted in improving CF properties at the microstructural level, explores the ensuing impact on mechanical, thermal, electrical, and photocatalytic properties.

The primary objective of this investigation is to scrutinize the structural, morphological, and catalytic attributes of CF composites featuring MWCNTs and FNDs as double hybrid nano-reinforcements. By comparing CF/MWCNTs, CF/MWCNTs-FNDs, and pure CF samples, the study aims to elucidate the impact of these nanofillers on the properties of CF composites. Additionally, the paper aims to contribute to the scientific understanding of the photocatalytic activity of CF samples, particularly in the degradation of Alizarin red (AR) dye.

The contributions of this work are multifaceted. Firstly, the paper delves into the structural and morphological characterizations of CF composites containing MWCNTs and FNDs, emphasizing a comparative analysis with pure CF samples. Noteworthy is the demonstration that including nanofillers, particularly FNDs, enhances the microstructure, pore size, and dispersion of CF composites, leading to improved mechanical, thermal, and electrical attributes. The best performance is observed in the CF/MWCNTs-FNDs hybrid samples. Furthermore, the study explores the photocatalytic activity of CF samples against AR dye, elucidating the synergistic effects of MWCNTs and FNDs in achieving a remarkable decolorization rate of approximately 88%.

The employed methods encompass the synthesis of pretreated CF pitches using coal tar pitch, followed by forming, carbonization, and graphitization techniques. The structural and morphological properties of CF composites are characterized through scanning electron microscopy (SEM) and energy-dispersive X-ray (EDX) analysis, confirming elemental composition. X-ray diffraction (XRD) analysis verifies the crystalline structure, while thermal conductivity is measured using Laser Flash Technology. The investigation of photocatalytic activity against AR dye involves repeated measurements to ensure the reliability and accuracy of the results.

The results underscore the success of incorporating MWCNTs and FNDs into the CF matrix, leading to improved structural and morphological properties. The CF/MWCNTs-FNDs hybrid composites exhibit superior thermal, mechanical, and electrical properties, with remarkable compressive strength, electrical conductivity, and thermal conductivity. Moreover, the photocatalytic activity of CF/MWCNTs-FNDs composites, particularly with 4 wt% loading, stands out with an 88% decolorization rate of AR dye. This decolorization is influenced by catalyst dosage, pH, and initial dye concentration.

In conclusion, the study demonstrates that incorporating MWCNTs and FNDs into CF composites significantly enhances their mechanical, electrical, thermal, and photocatalytic properties. The findings contribute to understanding hybrid nanomaterial reinforcements in carbon foam, offering insights for applications demanding superior multifunctional properties.

#### 5.3.9. Partial Conclusions

Table 4 provides an expansive view of how microstructural elements critically influence the functional attributes of nanocomposites. This compilation of research highlights the integral role of microstructure in defining these materials’ mechanical, optical, electrical, and biomedical properties.

Critical insights from these studies demonstrate that microstructural considerations, such as crosslinking time and the incorporation of specific nanostructures, are pivotal in determining the overall performance of nanocomposites. Advanced analytical methods, including UV–Vis analytical spectroscopy, have been employed to explore these materials’ antimicrobial properties, offering new perspectives on their potential applications.

The significance of pure carbon fibers within the architecture of nanocomposites is particularly noteworthy, underlining their essential contribution to enhancing the materials’ overall properties. These fibers represent a critical component in the development of advanced nanocomposite systems.

The array of research presented in the table underscores the interdisciplinary nature of nanocomposite science, bridging gaps between various scientific domains. This interconnection is crucial in the evolution of scientific inquiries and the development of innovative solutions.

In the biomedical field, applying laser-synthesized magnesium nanoparticles and other graphene-based nano-functional materials illustrates the transformative potential of nanocomposites. These materials have shown promising results in applications ranging from tissue engineering to polymer science and structural engineering.

Integrating machine learning techniques with nanomaterials research is also highlighted, showcasing an emerging synergy that could lead to breakthroughs in material science and other related fields.

The research on ZnO:GO/rGO composite thin films reveals promising structural, optical, and electrical properties, making them suitable candidates for energy harvesting applications, particularly in photovoltaic technology and dye-sensitized solar cells.

The effectiveness of MnNiWO_4_/CNF hybrid nanocomposites in photocatalytic dye degradation demonstrates their potential in advancing water treatment technologies, marking a significant step toward sustainable solutions.

Future research should explore the intersection of nanotechnology with various scientific disciplines to address contemporary challenges and advance the field of nanocomposite research. Investigations into the photocatalytic behavior of carbon foam composites, particularly for dye degradation, and the electrical properties of nanocomposites for energy devices remain areas of high potential.

The exploration of graphene-based materials in zinc oxide thin films for energy-related applications and the enhancement of carbon foam composites with MWCNTs and FNDs at the microstructural level present promising avenues for future study. Additionally, the potential of graphene-based surface modifications in bio-based composites, specifically with wheat straw, opens up opportunities for developing sustainable, high-performance materials.

### 5.4. Results Overview

This study has significant practical implications across various technological domains, particularly highlighting nanocomposites’ increasing recognition and potential applications in sectors such as microelectronics, optoelectronics, power transmission, and optoelectronic devices.

It offers valuable insights into the intricate interplay between nanocomposites’ material composition, structure, and functionality, thereby facilitating the development of materials tailored to specific technological requirements.

Furthermore, a meticulous analysis of crosslinking time’s impact on nanocomposite attributes underscores the critical importance of optimizing crosslinking degrees. This optimization can result in fine-tuning electrical properties and improved durability in power systems, thereby delivering tangible benefits in technological applications.

Integrating graphene nanoparticles into aluminum conductors is a promising avenue for enhancing their mechanical and electrical properties, especially for high-capacity power transmission. Investigating the fatigue behavior of nanocomposites at elevated temperatures holds the potential for addressing challenges related to fatigue failure in commercial aluminum conductors.

Moreover, the study suggests a promising direction for future research involving synthesizing and characterizing CdO:Sn nanocomposites using laser ablation. These endeavors deepen our understanding of these materials’ structural, optical, and electrical properties, potentially opening new avenues for technological advancement.

Beyond technological applications, this research underscores the versatility of nanocomposites, spanning diverse domains, including eco-friendly and biomedical applications. Notably, they promise improved dental healthcare by being employed in denture materials.

In light of these findings, future research should prioritize optimizing crosslinking degrees in nanocomposites, investigating the impact of water absorption on performance, and devising strategies to mitigate wear rates and friction coefficients while preserving enhanced material properties.

Concurrently, the study presents an extensive bibliometric and sentiment analysis of nanocomposite literature, revealing evolving trends and heightened recognition within the academic discourse.

A thematic analysis of the Scopus database underscores the substantial emphasis on microstructural studies in nanocomposite research, highlighting the significance of understanding microscopic organization and its correlation with material properties.

Moreover, focused attention on the electrical properties of nanocomposites sheds light on their critical attributes and intricate relationship with microstructure.

Furthermore, the analysis emphasizes the necessity of investigating nanocomposites’ mechanical behavior, especially their responses to varying stress conditions.

Notably, sentiment analysis of abstracts reveals a gradual shift toward more positive sentiment over time, indicative of researchers’ improved articulation of the significance of their work or enthusiasm for their findings.

In summary, this study significantly contributes to a deeper understanding of nanocomposite research themes, fosters interdisciplinary collaborations, and underscores the expanding interest in nanocomposites across various fields.

## 6. Conclusions

The analysis of bibliometric data concerning nanocomposites, with a focus on their electrical attributes, unveils significant patterns and novel areas worthy of attention. The significance of crosslinking duration in defining nanocomposite performance stands out, pinpointing it as a prime subject for subsequent exploration. Delving deeper into the nexus between microstructure and electrical properties while fine-tuning crosslinking variables emerges as imperative.

The sentiment analysis uncovers a favorable research ambiance, implying effective communication of findings within the research community. It is advisable for upcoming research endeavors to adopt a long-term sentiment analysis strategy, correlating sentiment trajectories with technological landmarks and policy modifications.

The exploration into cooperative research accentuates the vital role of bridging various disciplines. Crafting a structured approach for these interdisciplinary liaisons through digital channels can elevate the cumulative research efficacy and real-world applications.

However, this investigation has its limitations. A predominant reliance on the Scopus database and the literature penned in English presents certain boundaries. It is recommended that subsequent inquiries broaden their scope, incorporating varied databases and embracing literature from multiple languages to capture a more comprehensive global snapshot.

Venturing into domains like cutting-edge characterization methodologies, applications in the medical sphere, integration with optoelectronics, and the development of conductive nanocomposites holds promise. Channeling efforts into these arenas, with a spotlight on materials amplified by nanoparticles, can propel progress in vital sectors such as healthcare and sustainable energy solutions.

To encapsulate, this inquiry enriches the comprehension of the nanocomposite research landscape, steering the direction of future academic pursuits. The ramifications of this study span both technological evolution and broader societal advancements, underscoring the premise that sustained growth and collaboration in this domain are pivotal for bolstering global technological prowess.

The investigation conclusively demonstrates the critical role of microstructure, electrical, and mechanical properties in nanocomposites, aligning with pre-established expectations without significant deviations. This corroboration solidifies the methodology’s validity and sets a course for multidisciplinary research expansion, underpinning the study’s foundational strength and forward momentum in nanocomposite science. Adopting fuzzy logic and neural networks for subsequent research is recommended, recognizing the intricate dynamics within nanocomposite studies. These advanced computational approaches will refine analytical depth by assimilating varied data forms, thereby deepening the understanding of nanocomposite attributes. Emphasizing cross-disciplinary collaboration, enhanced computational strategies, and an expanded database and linguistic spectrum, the study aims to streamline research methodologies, augment efficiency in scientific exploration, and widen the accessibility of intricate domains. Future endeavors will quantitatively evaluate these methodologies, aiming to substantiate their efficacy in optimizing research productivity and reducing costs, further endorsing the imperative of such sophisticated techniques in fostering technological innovation and societal progress.

## Figures and Tables

**Table 1:** Holistic view of the nanocomposite research landscape.

Section	Key Insights	Recommended Keywords
**4.1. Scopus Database Search Strategy**	- Prominence in microstructural research. - Historical trends peaking between 2018 and 2022. - Post-2022 decline, indicating potential research saturation.	- Microstructure in Nanocomposites - Electrical Properties of Nanocomposites - Mechanical Behavior of Nanocomposites - Nanocomposite Films - SEM in Nanocomposite Research
**4.2. Textual Analytics Approach**	- Importance of collaborations in innovation. - Emerging trends in electromagnetic interference and sustainable materials. - Rise of Chinese language in studies.	- Nanocomposite Collaborations - Highly Cited Nanocomposite Articles - Emerging Trends in Nanocomposites - Global Funding in Nanocomposites - Language Trends in Nanocomposite Research - Sentiment Analysis in Nanocomposite Abstracts
**4.3. Scholarly Literature Analysis**	- Diverse themes, including analysis, synthesis, and performance enhancement. - Growing focus on biomedical applications and optoelectronic properties.	- Nanocomposite Research Themes - Advanced Characterization Techniques - Biomedical Nanocomposites - Optoelectronic Nanocomposites - Thin-Film Nanocomposites - Conductive Nanocomposites - Structural Analysis of Nanocomposites - Nanoparticle-Enhanced Materials - High-Performance Nanocomposites
**4.4. Analysis of MAP and NET Files**	- Strong correlation (R^2^ = 0.998) between term frequency and Total Link Strength (TLS). - Identification of thematic clusters and temporal trends.	- Bibliometric Network Analysis Themes - Total Link Strength (TLS) - Occurrences - Outliers in Bibliometric Analysis - Evolutionary Stage of Research Domains - Thematic Clustering in Materials Science - Temporal Trends in Research Topics - Emerging Research Trends - Interconnectedness of Terms - Research Themes in Optics and Materials Science
**4.5.1. Nanocomposites and Electrical Properties**	- Relationships between nanocomposites and electrical properties. - Shift toward investigating electrical attributes of nanocomposites.	- Nanocomposites - Electrical Properties - Microstructure - Crosslinking Time - Optoelectronic Behaviors - Interdisciplinary Research - Fundamental Mechanisms - Materials Science - Electrical Engineering - Analytical Techniques
**4.5.2. Nanocomposites and Mechanical Behavior**	- Interplay between microstructure and nanocomposite properties. - Exploration of multifunctional nanocomposites in various sectors.	- Nanocomposites - Mechanical Behavior - Microstructure - Nanoparticles - Crosslinking Time - Multifunctional Nanocomposites - Advanced Analytical Techniques - Energy Storage - Optoelectronics - Biocompatible Nanocomposites
**4.5.3. Nanocomposites and Microstructure**	- Detailed analysis of microstructure in nanocomposites. - Focus on nanocomposite fibers and optical properties.	- Nanocomposites - Microstructure - Crosslinking Time - Optical Properties - Energy Storage - Spectroscopic Techniques - Nanocomposite Fiber - Nanocomposite Material - Microstructural Features - Molybdenum - PBAT Nanocomposite - Al_2_O_3_ Tin Nanocomposite - Zinc Oxide–Nickel Oxide - Carbon Nanofiber Polystyrene Nanocomposite - UV–Vis Analytical Spectroscopy

**Table 2:** Synoptic summary regarding nanocomposites and electrical properties.

Subsection	Key Insights	Specific Studies and Findings
**5.1.1. XLPE/OMMT Nanocomposites**	Crosslinking degree significantly affects mechanical and electrical properties.	Yunzi et al. (2023) [465]: Utilized XRD, SEM, and gel content test to demonstrate the influence of crosslinking on XLPE/OMMT nanocomposites’ tensile and dielectric properties.
**5.1.2. PVDF/PMMA/BaTiO_3_ Nanocomposites**	BaTiO_3_ nanofillers’ impact on polymer blend nanocomposites for optoelectronic applications.	Sengwa et al. (2023) [466]: Analyzed the effects of BaTiO_3_ concentration on the properties of PBNC films using SEM, XRD, and FTIR.
**5.1.3. Aluminum–Graphene Nanocomposites**	Enhancing fatigue life in nanocomposites for power transmission.	Azizi et al. (2023) [467]: Investigated high-cycle fatigue in Al-0.5 wt% GNP composites, employing quasi-static and fatigue tests.
**5.1.4. XLPE/OMMT Water-Tree Aging**	Crosslinking degree’s effect on water-tree aging in nanocomposites.	Dong et al. (2023b) [468]: Conducted accelerated water-tree aging tests on XLPE/OMMT nanocomposites to study the effect of crosslinking.
**5.1.5. CdO:Sn Nanocomposites**	Effects of Sn doping in laser ablated nanocomposites for nanophotonics.	Fadhali (2023) [469]: Synthesized CdO:Sn nanocomposites to explore their structural, optical, and electrical properties.
**5.1.6. ZnO/TiO_2_ Nanocomposites in PEO/CMC**	Application of nanoparticles in bionanocomposites for flexible optoelectronics.	Ragab (2023) [470]: Analyzed PEO/CMC nanocomposites incorporated with ZnO/TiO_2_ NPs for optoelectronic technologies.

**Table 3:** Synoptic summary of studies on nanocomposites and mechanical behavior.

Subsection	Key Insights	Specific Studies and Findings
**5.2.1. WE43 Magnesium-Based Nanocomposites**	Improvement of mechanical and antibacterial properties through Friction Stir Processing (FSP).	O. Esmaielzadeh et al. (2023) [471]: Demonstrated enhanced strength and antibacterial properties of WE43 magnesium alloy using ZnO and CuZnO particles through FSP.
**5.2.2. XLPE/OMMT Nanocomposites**	Impact of crosslinking degree on mechanical and electrical properties.	Gao Dongyunzi et al. (2023) [465]: Analyzed how crosslinking affects XLPE/OMMT nanocomposites using XRD, SEM, and gel content tests.
**5.2.3. Al_2_O_3_/Brass Matrix Nanocomposites**	Al_2_O_3_ nanoparticle reinforcement’s effect on mechanical properties and wear behavior.	Shayan Memar et al. (2023) [472]: Studied the mechanical properties of Al_2_O_3_-reinforced brass matrix nanocomposites using stir casting.
**5.2.4. ZnO Nanoparticles in PLA/PCL Bionanocomposites**	Incorporation of ZnO nanoparticles improves structural, thermal, mechanical, and biocompatible properties.	Amir Babaei et al. (2023) [473]: Examined PLA/PCL bionanocomposites containing ZnO nanoparticles, focusing on their structural, thermal, mechanical, and biocompatibility aspects.
**5.2.5. Aluminum Oxyhydroxide in Dental Nanocomposites**	Enhancement of mechanical and tribological properties in dental resin composites with AlOOH.	Savita Kumari et al. (2023) [474]: Investigated the addition of aluminum oxyhydroxide to resin composites, improving mechanical properties for dental applications.

**Table 4:** Synoptic summary of studies on nanocomposites and microstructure.

Subsection	Key Insights	Specific Studies and Findings
**5.3.1. PDLC Films with MWCNT-Loaded Reticular Nanofiber**	Enhancement of electro-optical properties in PDLC films using MWCNT-loaded nanofibers.	Miao et al. (2023) [475]: Investigated improved electro-optical properties of PDLC films by optimizing the interaction between MWCNTs and PDLC using reticular nanofiber films.
**5.3.2. Zinc Oxide Nanorods in Cs/PVP Polymers**	Structural, optical, thermal, and electrical enhancement of nanocomposites using ZnO nanorods with Cs/PVP.	Alghamdi and Rajeh (2023) [476]: Analyzed the synergistic effects of ZnO nanorods in Cs/PVP polymer blends for potential applications in energy storage and thin-film solar cells.
**5.3.3. High-Entropy Nanofibers in Polymer Composites**	Transformation of energy storage performance in polymer composites using high-entropy nanofibers.	Dou et al. (2023) [477]: Introduced high-entropy nanofibers to improve dielectric breakdown properties and cyclic charge–discharge reliability in polymer composites.
**5.3.4. MnNiWO_4_ on Carbon Nanofiber for Photocatalytic Dye Removal**	Efficient photocatalytic dye removal using MnNiWO_4_ nanostructures on carbon nanofibers.	Sai Kumar A. et al. (2023) [478]: Synthesized MnNiWO_4_ hybrid nanostructures on CNFs for photocatalytic removal of dyes under light illumination.
**5.3.5. ZnO:GO/rGO Composite Thin Films for Energy Harvesting**	Synthesis and characterization of GO and rGO with ZnO for advanced energy harvesting applications.	Joshi et al. (2023) [479]: Explored the impact of GO and rGO on ZnO thin films, assessing their potential in photovoltaic technology and DSSCs.
**5.3.6. Magnesium Nanoparticles for Tissue Engineering**	Production and application of magnesium nanoparticles for tissue engineering and biochemical reactions.	Nyabadza et al. (2023) [480]: Produced MgNPs using laser ablation techniques for tissue engineering applications, enhancing cell growth and biochemical reactions.
**5.3.7. Bio-Based PLA Composites with GBMs and Wheat Straw**	Enhancement of bio-based PLA composites using graphene-based materials and wheat straw.	Chougan et al. (2023) [481]: Investigated the use of GBMs for surface functionalization of wheat straw to improve the mechanical and thermal performance of PLA bio-based composites.
**5.3.8. Carbon Foam with MWCNTs and FNDs**	Improvement of carbon foam properties with the addition of MWCNTs and functionalized nanodiamonds.	Aslam et al. (2023) [482]: Explored the enhancement of pitch-derived carbon foam through MWCNT and FND integration, focusing on mechanical, thermal, electrical, and photocatalytic properties.

## Data Availability

The raw data files are available here: https://github.com/ftir-mc/Nano-MEM, accessed on 25 October 2023.

## Supplementary Materials

The following supporting information can be downloaded at: https://www.mdpi.com/article/10.3390/ma17051088/s1, Figure S1: Methods and results.

## Abbreviations

AFM	Atomic Force Microscopy
AI	Artificial Intelligence
Al_2_O_3_	Aluminum Oxide
BET	Brunauer–Emmett–Teller theory
BM	Ball Milling
BM	Ball Milling
CF	Carbon Fiber or Carbon Foam
CP	Catalytic Performance
CSV	Comma-Separated Values
DE	Euclidean Distance
DNA	Deoxyribonucleic Acid
DOCTYPE	Document Type
DOCX	Microsoft Word Document File Format
DOI	Digital Object Identifier
Dye	Organic compound used for coloring
EC	Electrocatalyst
EDS	Energy-Dispersive X-ray Spectroscopy
EDX	Energy-Dispersive X-ray
EM	Electron Microscopy
EMI	Electromagnetic Interference
ENR	Epoxidized Natural Rubber
ENR	Epoxidized Natural Rubber
FESS	Field Emission Scanning Electron Spectroscopy
FT-IR or FTIR	Fourier Transform Infrared Spectroscopy
H_2_O_2_	Hydrogen Peroxide
HN	Hybrid Nanocatalyst
LDA	Latent Dirichlet Allocation
LSBI	Link Strength Between Items
LST	Low Shear Stress
MAP file	Visualization file format used by VOSviewer
MWCNT	Multiwalled Carbon Nanotube
NaN	Not a Number
NET file	Network file format used by VOSviewer
NiO	Nickel Oxide
NiWO_4_	Nickel Tungstate
NLP	Natural Language Processing
NLTK	Natural Language Toolkit
NMR	Nuclear Magnetic Resonance
ORCID	Open Researcher and Contributor ID
PA	Photocatalytic Activity
PBAT	Polybutylene Adipate Co-terephthalate
PBVS	Python Boosted Visualization of Similarities
PMMA	Polymethyl Methacrylate
PN	Polymer Nanocomposite
PP	Polypropylene
Pt	Platinum
PU	Polyurethane
R^2^	Coefficient of Determination
RIS	Research Information Systems File
RMSE	Root Mean Squared Error
ROS	Reactive Oxygen Species
Scopus	A bibliographic database for academic research
SEM	Scanning Electron Microscopy
TEM	Transmission Electron Microscopy
TITLE-ABS-KEY	Search for terms only in Titles, Abstracts, and Keywords in the Scopus database
TLS	Total Link Strength
TXT	Text File
UV	Ultraviolet
UV–Vis	Ultraviolet–Visible Spectroscopy
UV–Vis Analytical Spectroscopy	Ultraviolet–Visible Analytical Spectroscopy
VL	Visible Light
VOSviewer	Visualization of Similarities Viewer
XLPE	Crosslinked Polyethylene
XPS	X-ray Photoelectron Spectroscopy
XRD	X-ray Diffraction
XRF	X-ray Fluorescence
ZnO	Zinc Oxide
ZOId	Diameters of Zone of Inhibition
ZOI	Zone of Inhibition
