# Supplementary figures and images for: A 30-Year Review on Nanocomposites: Comprehensive Bibliometric Insights into Microstructural, Electrical, and Mechanical Properties Assisted by Artificial Intelligence
Source: Materials (Basel). 2024 Feb 27;17(5):1088. doi: 10.3390/ma17051088 (PMC10934506; doi:10.3390/ma17051088)

# Structure of the Methods

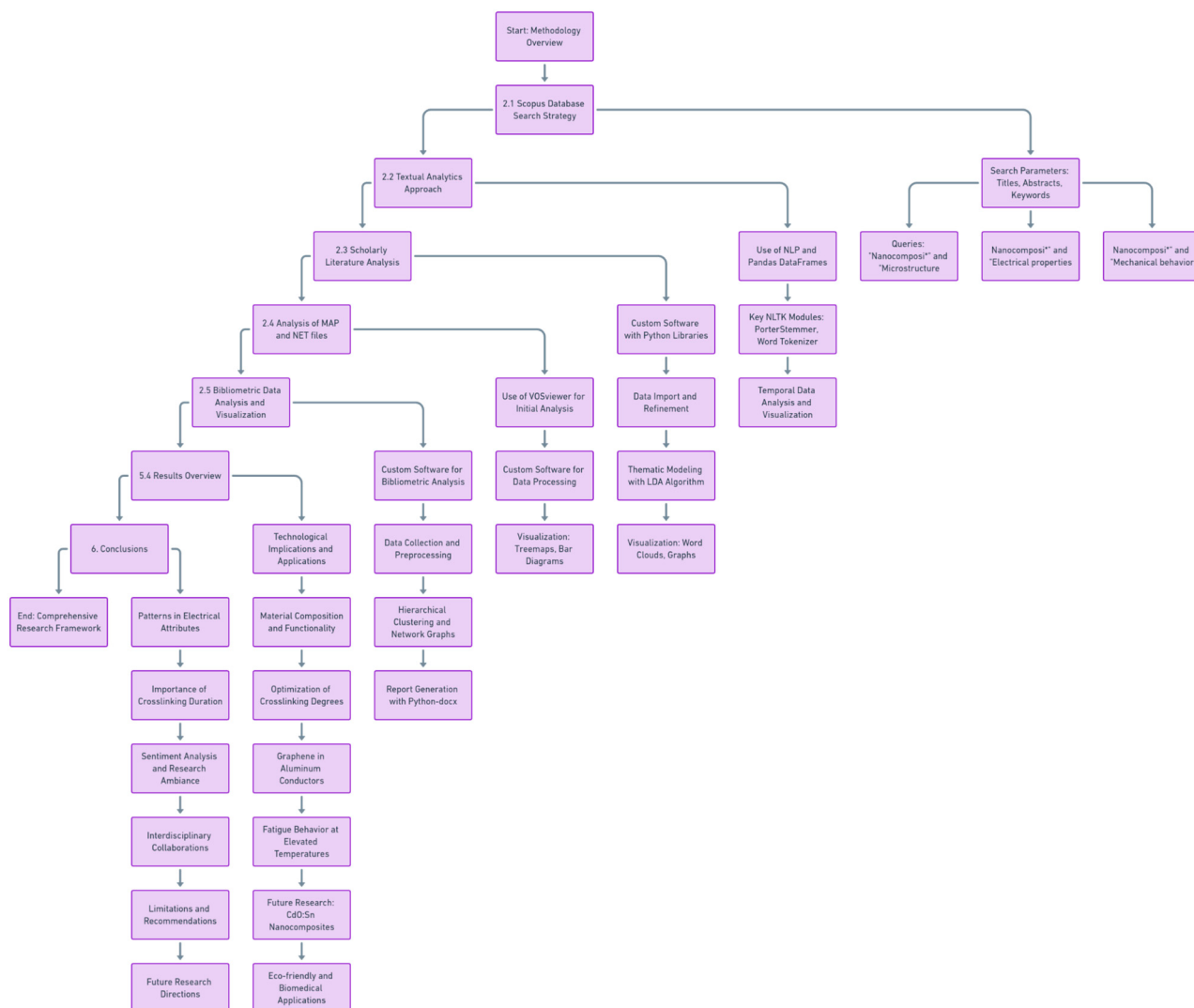

Made with 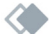 Whimsical

Figure S1. Methods and results.

Supplement: Supplementary file 1 [file materials-17-01088-s001.zip › materials-2828318-supplementary.pdf]
